# Bushmeat hunting and extinction risk to the world's mammals

**DOI:** 10.1098/rsos.160498

**Published:** 2016-10-19

**Authors:** William J. Ripple, Katharine Abernethy, Matthew G. Betts, Guillaume Chapron, Rodolfo Dirzo, Mauro Galetti, Taal Levi, Peter A. Lindsey, David W. Macdonald, Brian Machovina, Thomas M. Newsome, Carlos A. Peres, Arian D. Wallach, Christopher Wolf, Hillary Young

**Affiliations:** 1 GlobalTrophic Cascades Program, Department of Forest Ecosystems and Society, Oregon State University, Corvallis, OR 97331, USA; 2 Forest Biodiversity Research Network, Department of Forest Ecosystems and Society, Oregon State University, Corvallis, OR 97331, USA; 3 Department of Fisheries and Wildlife, Oregon State University, Corvallis, OR 97331, USA; 4 School of Natural Sciences, University of Stirling, Stirling FK9 4LA, UK; 5 Institut de Recherche en Ecologie Tropicale, CENAREST, BP 842 Libreville, Gabon; 6 Grimsö Wildlife Research Station, Department of Ecology, Swedish University of Agricultural Sciences, 73091 Riddarhyttan, Sweden; 7 Department of Biology, Stanford University, Stanford, CA 94305, USA; 8 Universidade Estadual Paulista (UNESP), Instituto Biociências, Departamento de Ecologia, 13506-900 Rio Claro, São Paulo, Brazil; 9 Department of Bioscience, Ecoinformatics and Biodiversity, Aarhus University, 8000 Aarhus, Denmark; 10 Panthera, 8 West 40th Street, 18th Floor, New York, NY 10018, USA; 11 Mammal Research Institute, Department of Zoology and Entomology, University of Pretoria, Pretoria, Gauteng, South Africa; 12 Wildlife Conservation Research Unit, Department of Zoology, University of Oxford, The Recanati-Kaplan Centre, Tubney House, Tubney, Abingdon OX13 5QL, UK; 13 Department of Biological Sciences, Florida International University, Miami, FL 33199, USA; 14 School of Life and Environmental Sciences, Centre for Integrative Ecology, Deakin University, Burwood campus, Geelong, Victoria 3125, Australia; 15 School of Life and Environmental Sciences, The University of Sydney, New South Wales 2006, Australia; 16 School of Environmental and Forest Sciences, University of Washington, Seattle, WA 98195, USA; 17 School of Environmental Sciences, University of East Anglia, Norwich NR4 7TJ, UK; 18 Centre for Compassionate Conservation, School of Life Sciences, University of Technology Sydney, PO Box 123 Broadway, New South Wales 2007, Australia; 19 Department of Ecology and Evolutionary Biology, University of California Santa Barbara, Santa Barbara, CA 93106, USA

**Keywords:** wild meat, bushmeat, hunting, mammals, extinction

## Abstract

Terrestrial mammals are experiencing a massive collapse in their population sizes and geographical ranges around the world, but many of the drivers, patterns and consequences of this decline remain poorly understood. Here we provide an analysis showing that bushmeat hunting for mostly food and medicinal products is driving a global crisis whereby 301 terrestrial mammal species are threatened with extinction. Nearly all of these threatened species occur in developing countries where major coexisting threats include deforestation, agricultural expansion, human encroachment and competition with livestock. The unrelenting decline of mammals suggests many vital ecological and socio-economic services that these species provide will be lost, potentially changing ecosystems irrevocably. We discuss options and current obstacles to achieving effective conservation, alongside consequences of failure to stem such anthropogenic mammalian extirpation. We propose a multi-pronged conservation strategy to help save threatened mammals from immediate extinction and avoid a collapse of food security for hundreds of millions of people.

## 1. Introduction

Rapid loss of biodiversity in recent times indicates that a sixth mass extinction event is underway on the Earth, whereby the average rate of vertebrate species loss is now up to 1000 times higher than background rates [1]. Population sizes of vertebrate species have been declining, mainly due to the twin threats of direct exploitation and habitat destruction [2,3]. Unsustainable hunting for consumption and trade of wild meat (also known as bushmeat) by humans represents a significant extinction threat to wild terrestrial mammal populations, perhaps most notably in parts of Asia, Africa and South America [4–6]. Here, we refer to predominantly unregulated (and often illegal and unsustainable) harvesting of wildlife for human consumption as ‘bushmeat hunting’ or ‘wild meat’ hunting. This is distinguished from legal or regulated hunting of wildlife which can be sustainable. This global bushmeat hunting crisis is a fundamentally distressing problem to address because it is intimately tied to human development challenges such as food insecurity, emergent disease risks and land-use changes [7]. While many ethnic groups have hunted wildlife for subsistence over millennia, often with highly detrimental effects [8], the unsustainablility of this practice has accelerated in many areas due to growing human populations, an increasing tendency for wild meat to be traded commercially [9], and the widespread adoption of firearms and motorized transport that increase the efficiency and spatial extent of hunting [10,11]. Larger species are typically targeted by bushmeat hunters first and are also the least able to bear hunting offtakes [12–14]. As wildlife populations outside protected areas decline, poaching pressure is increasing in many parks and reserves. As a consequence many forests, savannahs, grasslands and deserts in the developing world are now becoming ‘empty landscapes’ [14] devoid of harvest-sensitive wild mammals [15–17].

Herein, we present the first comprehensive global assessment of hunting on the world's terrestrial mammals, and provide new data and insights based on our analysis (box 1). We report on the species most severely threatened by bushmeat hunting and review trends in their endangerment status. We analyse the geographical distributions of critically overhunted mammals and determine the extent to which their ranges are protected. We highlight how spatially and taxonomically biased research efforts have impeded recognition of both the declines of smaller or less iconic species and also the importance of their ecological roles. In some cases, these species may already be ecologically defunct with ecosystem interactions virtually absent. We discuss growing evidence that ecological functionality cannot necessarily be replaced, leading to cascading alterations of ecosystems, the loss of ecological interactions and the loss of other ecosystem and social services [18,19]. We conclude by outlining pathways to better understand human predatory behaviour and we provide a conservation plan for the twenty-first century. Only bold changes will substantially diminish the imminent possibility of humans consuming many of the world's wild mammals to the point of functional [20] or global [19,21] extinction.

Box 1. **Overview.**

Our assessment revealed the following:

— This is the first global assessment of mammal hunting and results show evidence of a global crisis.
— 301 terrestrial mammal species are threatened with extinction due to hunting by humans. All of these species occur in developing countries.
— Orders with the most species threatened by hunting include primates, even-toed ungulates, bats, diprotodont marsupials, rodents and carnivores.
— The primary reason for hunting and trapping these mammals is to acquire meat for human consumption, medicinal products, ornamental use and pet trade.
— The likelihood of extinction threat of heavily hunted mammals is proportional to body size.
— Only 2% of the mammals threatened by hunting have populations considered stable or increasing.
— These heavily hunted mammal species have, on average, only 10.5% of their ranges within protected areas.
— Regions with the most species threatened by hunting include Asia (especially SE Asia) and Africa.
— Countries with the most endemic species threatened by hunting include Madagascar, Indonesia, Philippines, Brazil, Papua New Guinea, India and China.
— Species affected by severe hunting are also often victims of habitat deterioration, expanding agriculture, human settlement encroachment and livestock competition.
— Through cascading effects, the loss of these mammals is altering the structure and function of the environments in which they occur, and this could result in a loss of food security for humans.
— Based on the number of published articles, there is a research bias favouring the heavier species and those found in Africa.
— There has been little conservation progress in reversing the fate of these threatened mammals despite several major summits convened on biodiversity conservation and protected areas.

## 2. Severe impacts of hunting on mammal species

**Figure 1.**
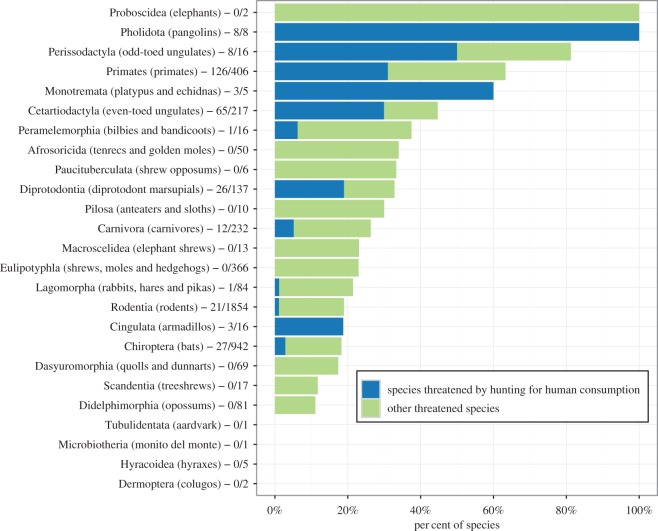
The percentage of species threatened by hunting for human consumption and other threatened species in each mammalian order. The values on the *x*-axis refer to the percentage of species out of all mammal species in each order. The category ‘Other threatened species’ consists of the other threatened mammal species where hunting for consumption is not a primary or major threat. Horizontal bars are sorted from highest to lowest total percentage of threatened species in each order. Numbers on the *y*-axis after the order names are the number of species threatened by hunting followed by the total number of species in the order. Elephants are threatened by hunting but not listed here because they are predominately killed for their ornamental ivory and not for the consumption of meat or medicine (see [14]). The order Notoryctemorphia (marsupial moles) was omitted as it contains only data-deficient species.

**Figure 2.**
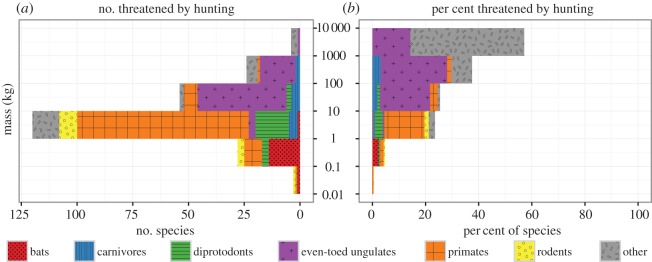
Number (*a*) and percentage (*b*) of mammal species threatened by hunting grouped by body mass in kilograms. The values correspond to species threatened by hunting out of all terrestrial mammals in each body mass group, respectively. Body mass data come from [23–25].

According to the International Union for Conservation of Nature (IUCN), 1169 of the world's 4556 assessed terrestrial mammals (approx. 26%) are listed as threatened with extinction [22]. Here, using individual species accounts from the IUCN Red List, we identify 301 threatened mammal species for which a primary threat is hunting by humans (see materials and methods in electronic supplementary material, table S1). This group of heavily hunted mammals represents 12 of the 26 extant terrestrial orders, approximately 7% of all assessed terrestrial mammals and approximately 26% of all threatened terrestrial mammal species on the Earth (figure 1). Endangerment categories for these 301 species include 115 vulnerable (VU = 38%), 114 endangered (EN = 38%) and 72 critically endangered (CR = 24%). Orders with the most species threatened by hunting include primates (126 species), even-toed ungulates (Cetartiodactyla, 65 species), bats (Chiroptera, 27 species), diprotodont marsupials (Diprotodontia, 26 species), rodents (Rodentia, 21 species) and carnivores (Carnivora, 12 species). Orders with the highest percentages of species threatened by hunting include pangolins (Pholidota, 100%), platypus and echidnas (Monotremata, 60%), odd-toed ungulates (Perissodactyla, 50%), primates (31%) and even-toed ungulates (30%). Mammal species threatened by hunting consist predominantly of ungulates for large-sized mammals (more than 10 kg), primates for medium-sized mammals (1–10 kg) and bats for small-sized mammals (less than 1 kg) (figure 2*a*).

### 2.1. Drivers of mammal hunting

The primary reason for hunting and trapping these 301 threatened mammal species is to acquire meat for human consumption (*n* = 285) and this occurs nearly entirely in developing countries across Africa, South America and particularly Southeast Asia (electronic supplementary material, figure S3). Other major reasons for hunting include consumption of body parts as traditional medicine (*n* = 67 species), live animals for the pet trade (*n* = 46) and ornamental uses of body parts (*n* = 36) (electronic supplementary material, figure S4). Species hunted for medicinal use primarily involves primates (*n* = 25) and ungulates (*n* = 25), but also various other taxa such as carnivores (*n* = 8) and pangolins (*n* = 8). Live trade mostly includes primates (*n* = 31), while ornamental uses (ivory, horns, antlers, skins, etc.) largely involve ungulate (*n* = 17), carnivore (*n* = 7) and primate (*n* = 6) species. Ornamental use includes animal parts from trophy hunts. Live trade and ornamental use of wild mammals occurs primarily in Southeast Asia and secondarily in Africa and Latin America (South America, Central America, Caribbean).

### 2.2. Distribution of impacts by body size

Mammal species threatened by hunting in the 1–10 kg mass group are the most numerous (*n* = 120) while those weighing 10–100 kg rank second (*n* = 54) compared with other body mass classes (figure 2*a*). However, considering the percentage of species within each mass category, the likelihood of threat is proportional to body size with a higher proportion of larger species particularly threatened by hunting, culminating in close to 60% of our largest terrestrial mammals (more than 1000 kg) being at risk of extinction from human consumption (figure 2*b*). Mammals threatened by hunting include species with a multitude of ecological roles from different levels of the food web, including apex predators, mesopredators, herbivores in all size classes, seed dispersal agents, pollinators and prey species.

### 2.3. Population trends of hunted species

Population trends of these species continue to worsen over time, with only 2% of these populations considered stable or increasing (electronic supplementary material, table S1 and S2). Between 1996 and 2008 (or later for more recently updated species), the conservation status of 23% of the heavily hunted mammal species deteriorated (63 of 270 species with data available), while only one species improved and the rest (*n* = 206; 76%) remained stable. However, 40 species were already classed as critically endangered by 1996, indicating there has been little or no conservation progress in reversing their fate since then despite several major summits convened on biodiversity conservation and protected areas (electronic supplementary material, table S2). Indeed, primates and even-toed ungulates had the highest number of species whose status deteriorated between 1996 and 2008 (23 and 16, respectively).

### 2.4. Geographical distribution of hunting impacts

**Figure 3.**
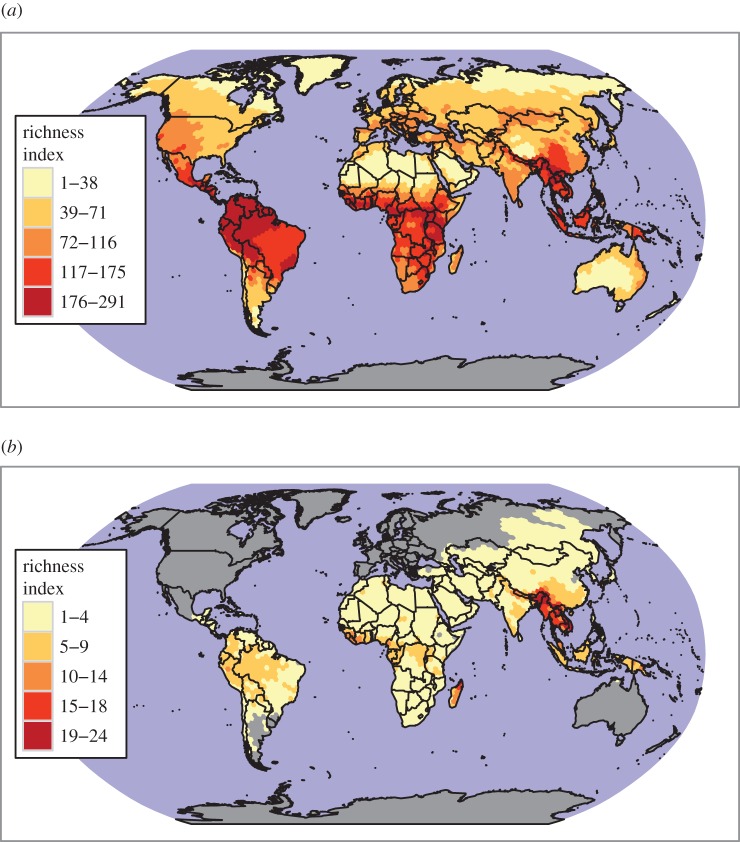
Species richness maps for (*a*) all terrestrial mammals and (*b*) mammals threatened by hunting.

The impact of mammal hunting on species endangerment differs among continents, and does not reflect closely parallel global patterns of mammal richness [26] (figure 3, S1). There are 113 species threatened by hunting in Southeast Asia (13% of all threatened mammals are east of India and south of China), 91 in Africa (8%), 61 in the rest of Asia (7%), 38 in Latin America (3%) and 32 in Oceania (7%) (figure 3, electronic supplementary material, figure S1). Notably, all of the 301 mammals that are threatened by hunting are found in developing countries, and only eight of these species are also found in developed countries, suggesting a huge contrast in dietary patterns, wildlife management and conservation efforts between the developing and developed world. However, part of this difference may be an artefact of humans extirpating megafauna in developed temperate regions during the Late Pleistocene [27]. Countries with the most endemic species (present in a single country) threatened by hunting include Madagascar (*n* = 46), Indonesia (*n* = 37), Philippines (*n* = 14), Brazil (*n* = 10), Papua New Guinea (*n* = 10), India (*n* = 6) and China (*n* = 5) (electronic supplementary material, figure S2).

Of the 301 hunted threatened species, three are already possibly extinct including the kouprey (*Bos sauveli*), Wondiwoi tree-kangaroo (*Dendrolagus mayri*) and little earth hutia (*Mesocapromys sanfelipensis*) (electronic supplementary material, table S1). The remaining 298 species have an average of 10.5% of their ranges within IUCN Class I–III protected areas and the majority (*n* = 162) have less than 5% of their ranges within protected areas (electronic supplementary material, figure S5 and table S3); 65 have ranges falling entirely outside protected areas.

### 2.5. Effects of unselective hunting methods

In some regions, wild meat is primarily obtained with traps and snares, but this is almost completely unselective and generates substantial bycatch [28]. In Central Africa, snare losses to scavengers and decomposition of the target species result in the wastage of up to a quarter of total captures [29]. This is also an important animal welfare problem, because up to one-third of animals escape with injury and unknown subsequent fates [29]. A study in Zimbabwe indicated that more than 1400 large mammals rotted and were wasted in snares over a 4-year period [30], with more animals rotting in snare-lines far from human settlements [31]. Unselective traps result in the capture of females and young, which confers much greater impacts for the many polygynous ungulate species than if harvests were restricted to adult males [17] (although for species that practise infanticide, such as bears and lions, killing males can be just as problematic). Even traditional forms of subsistence hunting can result in over-exploitation of particular species when human population density increases [32,33].

### 2.6. Additional factors exacerbating extinction risk

As hunting is facilitated by access to land [16] and the human footprint is increasing across the globe [34,35], species and regions affected by severe hunting are also often victims of habitat deterioration of many types including forest loss and fragmentation (*n* = 184 hunted species), expanding agriculture (*n* = 152), human settlement encroachment (*n* = 34) and livestock competition (*n* = 27) (electronic supplementary material, figure S6). Geographically these contributory factors vary; deforestation predominantly affects mammals of Southeast Asia, Central and West Africa, Madagascar, Amazonia and the Atlantic Forest of South America; agriculture and human settlement encroachment are major threats in Southeast Asia; and competition with livestock occurs across southern Asia, much of Africa and parts of South America (electronic supplementary material, figure S7).

## 3. Consequences of overhunting

Hunting and the trade of wild meat and body parts has long been recognized as a severe problem for a range of mammal species. This pervasive pressure on ecosystems can have wide-ranging effects that cascade beyond the loss of the hunted species, altering the structure and function of the environments in which they occur and the services they provide [36,37]. Wild meat is currently an important food source for humans worldwide. An estimated 89 000 metric tons of meat with a market value of approximately $200 million are harvested annually in the Brazilian Amazon [38], and exploitation rates of large mammals in the Congo basin are estimated to be five times higher than in the Brazilian Amazon [4]. With this trend, the loss of mammal populations thus affects the livelihoods and food security for hundreds of millions of rural people across the globe [39].

### 3.1. Cascading effects: large mammals

Mammals dominate the larger size classes of the modern animal kingdom and the severe overhunting we describe here is having a disproportionate impact on the largest species (figure 2*b*). This is contributing to dramatic global-scale population declines and range contractions for many large-bodied mammals [14,40–44]. As human hunters on all continents target mammals [45], and especially large-bodied ones, the ecological disruption of unsustainable human hunting is extensive—whether driven by subsistence or commercial hunting [46,47]. Since the Pleistocene, extinction has affected larger mammals more than smaller ones [19] and we suggest that contemporary extinction risk from overhunting is still a primary threat for the largest terrestrial mammals.

Large-bodied predators and herbivores provide ‘top-down’ control on ecosystems, which helps to balance the effects of environmental, or ‘bottom-up’ factors, such as primary productivity or climate. Their roles are crucial to ecosystem stability and their loss can result in particularly rapid, widespread and potentially irrevocable changes [13,14,18,48]. As no other taxonomic group contains terrestrial animals in the size classes of the large modern mammals, the functional loss of large mammals can rarely be compensated, leading to permanent ecosystem changes [49]. Even in the absence of targeted hunting, large predators may not survive competition with humans as their prey base is depleted by hunting [17,50,51]. Some large mammals perform non-redundant seed dispersal services for very large-seeded plants [52,53] and their local extirpation may lead to an increase in rodent populations and seed predation [54]. All of these examples highlight not only the importance of mammals for ecosystem functioning, but for ecosystem services to humans.

### 3.2. Cascading effects: small mammals

Hunting smaller mammals can also drive insidious alterations of ecosystems, as they can provide critical ecological functions including seed dispersal, consumption of vegetation and invertebrates, soil disturbance and prey items for other species. Of these functions, the role of smaller frugivorous mammals as seed dispersers, particularly primates and bats, has been the most clearly documented. Removal of these seed dispersers can initiate widespread changes in forest regeneration, composition and structure [55–57], and despite the overlap in their body size with birds and reptiles, studies show that the ecological role of mammals is not always replaceable by other taxa [58,59]. Likewise, some bat species serve as specialized pollinators and seed dispersers that are unlikely to be replaced. Yet bats are the largest group of mammals under 1 kg threatened by hunting [60].

Some smaller species are not targeted by human hunters (less than 1 kg, figure 2*a*) and as hunting removes the larger mammals from ecosystems, especially large herbivores that compete with smaller species [14], these small species can experience ecological release with some populations increasing substantially [54,61]. However, even super-abundant small prey cannot sustain large predators, as the energy required to catch and process multiple carcasses is not replaced because of their small biomass [62]. As such, the small mammal population remains unchecked, changing a wide range of species interactions and contact rates. The release of smaller mammals, particularly rodents, can become a dominant mechanism by which the effects of human hunting can cascade into transformation of invertebrate and plant communities [19].

### 3.3. Behavioural changes in target species

Mammals can employ complex predator avoidance strategies when faced with severe hunting pressure by humans. However, these survival strategies can have eco-evolutionary implications if direct costs are incurred from increased energy expenditure and reduced foraging time in human-mediated ‘landscapes of fear’ [62,63]. Changing hunter avoidance behaviours to become more nocturnal or more cryptic, can for example, lead to a reduction in hunting success for other predators competing with humans. Hunter avoidance that involves prey species moving to a perceived sanctuary area can also lead to conservation dilemmas [64]. In the last few decades, fencing of protected areas has been used as a tool to help control overhunting, in an attempt to reduce human incursions and prevent the movement of wildlife into unprotected landscapes. However, fencing restricts animal movements, reduces effective park sizes and destroys ecological connectivity [65,66].

### 3.4. Zoonotic diseases

The loss of large mammals, and in particular, the selective loss of primates, large ungulates and carnivores, may have strong consequences for zoonotic disease transmission. In the short term, high levels of contact with these species during the exploitation phase may increase transmission risk to humans. Hunting and butchering allow for high levels of direct contact of body fluids and are thought to have been important in emergence of Ebola, HIV-1 and -2, Anthrax, Salmonellosis, Simian foamy virus and other zoonotic diseases [67]. Given high rates of international trade in wild meat and human movement, this could easily have important short-term global health consequences [68]. However, in the longer run the extirpation and endangerment of these species will almost certainly result in the co-extinction of many parasites and pathogens, likely to include some potential zoonotic pathogens [69,70]. Yet, hunting in particular may continue to cause increases in zoonotic disease risk because of the selective nature of losses following hunting. The selective loss of large herbivores, for instance, is known to cause relatively systematic increases in abundance of rodents [71], which are thought to be particularly effective at hosting and transmitting human-borne zoonoses, thus driving landscape-level increases in rodent-borne disease [72]. Increases of rodents following large herbivore loss appear to be much more systematic than following other types of disturbance [73] probably due to generalized competitive release of herbivorous and granivorous rodents. This pattern may result in more rodent-borne diseases. Systematic predator loss may also tend to cause increases in disease risk, although mesopredator release could counter such a pattern in some systems [74]. There is also some evidence that smaller hosts, with faster life histories, tend to be more competent reservoirs for some common zoonoses, regardless of trophic cascades; as hunting favours these species both directly and indirectly, this may lead to systematic increases in zoonotic disease prevalence [75]. Certainly, smaller species are likely to be more abundant and in closer contact with humans, allowing more transmission opportunities.

### 3.5. Loss of food security for humans

The effects of unsustainable hunting are borne not just by the target species and their ecosystems, but, as unsustainability becomes apparent and hunting returns decline, by the hunter communities themselves. Our analysis shows that overhunting of threatened mammals is most common in developing countries, which is driving many of these mammals towards extinction. These countries by definition have poorer populations, generally lower food security than richer countries, and less capacity to deliver conservation [76]. Ensuring current food security without changing current hunting practices will inevitably fail to provide food security in years to come [39,77]. As hunters using unsustainable practices become unable to make ends meet, they may leave the hunting community and migrate [78], switch to less preferred species, increasing ecological impacts [45] or, with their families, begin to suffer malnutrition and critical poverty [77]. None of these scenarios are appealing as future situations for these people. In addition, large mammal communities offer potential to generate human food security with their income-generating capacity through tourism. Such potential is increasingly forgone as wildlife populations are overhunted for a much lower value product: meat [16].

## 4. Research and knowledge gaps

**Figure 4.**
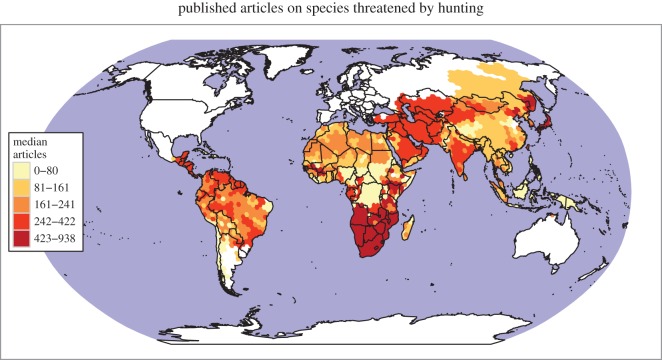
Research effort for mammal species threatened by hunting. The variable shown is the median number of articles published between 1965 and 2016 for all of the species threatened by hunting found in each hexagon (see text).

To achieve better conservation policies and practices for our surviving mammals, science must first provide a better platform for decision-making in identifying changes in wildlife populations and ecosystems. Our analysis of the mammals endangered by hunting shows a significant bias in the regions and species benefitting from research effort (figure 4). Many of the mammal species threatened by hunting remain poorly studied and are greatly in need of basic biological and ecological research, including simple evaluation of their remaining numbers. For example, some pangolin species may be approaching extinction in numerous areas due to hunting for medicinal products, but little is known about their remaining numbers [79]. Likewise, many populations are likely to have been already impacted when first assessed, leaving them vulnerable to the ‘shifting baseline’ effect, which prevents robust evaluation of the loss of their ecological role (electronic supplementary material, table S1).

Scientific research effort, as measured by the number of published articles on each species, has been relatively low in Southeast Asia (figure 4). The relative lack of research effort in this region is a major conservation issue as Southeast Asia has many more threatened and harvest-sensitive mammal species than most other regions (electronic supplementary material, figure S1). The general public prefers larger species [80], which also get more attention from researchers. Proportionally, research as depicted by the number of articles per threatened mammal species is positively correlated to species body mass (*p* < 0.001) (electronic supplementary material, figure S8). Therefore, designing sound conservation policies, or predicting the ecosystem effects of loss of the species with lower body masses, particularly outside Africa, is hampered. There is also a great need for comprehensive research on the world's birds and reptiles, which are often threatened by hunting, also with strong ecological and evolutionary consequences, but not covered in this review.

The geography of risk posed by hunting is likely to be biased by the fact that data for this study are derived from IUCN Red List data. These data are likely to place disproportionate emphasis on those areas which have long-standing problems with hunting, restricted species ranges and where there are multiple threats facing wildlife. Thus, our IUCN-derived spatial data on risks to mammals do not completely align with data on those areas that are experiencing extraordinarily high, and increasing, take in bushmeat such as in the Congo basin [4]. However, the ground-level data needed for comparative rates of harvest are unfortunately not available on a global scale.

Good conservation paradigms cannot be built only on ecological understanding; they require a sophisticated evaluation of the drivers of human behaviour and insight and innovation as to how that behaviour may be changed. Understanding the drivers of human behaviour is a key step to designing effective alternatives [81]. Research into human drivers of unsustainable harvesting is a relatively new field and more cross-disciplinary exchange is needed between ecologists, socio-economists and human behaviour specialists in order to tackle mammal over-harvesting.

## 5. Moving forward with five conservation actions

Growing human populations, increasing middle-class wealth, access to hunting technologies in developing nations and the modern ease of transporting goods around the planet are facilitating a global demand for wild animals as food and other products that simply cannot be met by current global wildlife populations. Furthermore, the growing penetration of remote landscapes by road networks is resulting in a lack of faunal refugia. The brunt of this overconsumption is borne by mammals, though many other taxa are also impacted (e.g. birds, fish, reptiles). In order to maintain viable and functional populations of these species, we must find ways to curb our unsustainable consumption of mammal species that are threatened or dramatically declining. In the absence of actions, the survival of wildlife will depend on the balance between supply and demand—to halt the over-exploitation of mammals we must work to stem demand through regulation, persuasion or provision of alternatives, and by increasing wildlife numbers through adequately enforced protection of existing populations and wild spaces. Science-based management of wildlife populations for sustained use can benefit both conservation and human livelihoods, but this is very difficult and requires the installation of expensive bureaucracies to monitor and manage hunting. For the preservation of these species, conservation research is essential. Below we suggest a five-pronged cross-disciplinary approach to reducing wildlife mortality and demand for wild meat (box 2). We briefly touch on some of the relevant considerations for these complex issues. Given space constraints, the following discussion is not exhaustive.

Box 2. **Five Conservation Steps.**

1. Increase legal protection of wild mammals by:
  (i) Creating clearer laws.
  (ii) Increasing penalties.
  (iii) Regulating formal harvests in some cases.
  (iv) Increasing regulatory support from developed countries.
2. Implement legal user rights for wildlife utilization
  (i) Empower local communities to capture the benefits from wildlife conservation with legal user rights over wildlife.
3. Provide alternative foods
  (i) Shift to hunting species resilient to hunting pressure.
  (ii) Provide incentives for communities to switch to traditionally grown protein-rich plant foods rather than wild meat.
4. Increase education and family planning
  (i) Support programmes helping to lower human birth rates, especially those in rural areas that enhance education opportunities for young women.
  (ii) Meet the need for family planning in areas where women want to avoid or delay pregnancy.
5. Change international policy
  (i) Wealthier nations must stop exacerbating the problem by inflating demand and price for meat, trophy, medicinal and ornamental products from wild mammals.
  (ii) Current attempts to reforming policies have been weak due to loopholes and poor governance. International agreements must include conventions to share the financial burden of responsibility among nations, especially the developed ones.

### 5.1. Legal protection of wild mammals

Enforcement of existing legal wildlife protection or improvement of legal instruments has a key role to play in reducing excessive harvests of wildlife in many contexts. Such solutions need to involve clearer laws governing the circumstances under which harvest of wildlife for meat is permissible, strong penalties for infringements, education of judiciaries and law enforcement. In some cases, outside of protected areas and for non-threatened species, regulation of formal legal harvests may provide a partial solution. However, developing countries often lack resources and the strength of governance to implement such regulation [76,82,83], so logistical and financial support from developed countries will be needed. Immediate protection for our most threatened mammals will also require functional protected areas with national support, professional staff, necessary funding and insightful governance [84]. Law enforcement to regulate hunting outside protected areas comprises multiple steps, including anti-poaching patrols, anti-trafficking controls designed to intercept wild meat during transit and regulation or suppression at points of sale. There is evidence, particularly from Africa, that well-funded and organized law enforcement can be effective at suppressing illegal hunting to sustainable levels [85]. But to achieve effective law enforcement substantially more funding is required than is available at present, with deficits being particularly acute in the tropics. Given the vast extent of protected area networks in many parts of the tropics, the size of their burden to the host countries and the scale of the threat from illegal hunting, there is a likely need for a significant future elevation in international funding for many parks to function effectively [86,87], and the costs of effective regulation outside protected areas are likely to be even higher [82]. Increasing funding and reducing corruption are ongoing and extremely difficult challenges.

### 5.2. Legal user rights for wildlife management

In many areas, particularly in Africa and South America, wildlife occurs on communal lands without clear ownership over land and wildlife resources, resulting in open access systems whereby it is advantageous for community members to harvest before someone else does [14]. Empowering local people to capture legal benefits from wildlife conservation can be an important step in reducing excessive illegal harvests, when efforts to provide alternative livelihoods are unsuccessful or at least inefficient levers for change in hunting behaviour [88]. Addressing the land rights of individuals within communities is an important step in avoiding the tragedy of the commons (actions of individuals depleting the common resource) [89]. The definition of the rights to resources at individual and community levels is a key prerequisite to empowering people to benefit legally from wildlife [90]. Wildlife can have a much higher value if used legally, either for tourism or sport hunting [30], and under the right conditions of land tenure and legislation, large quantities of legal wild meat can be produced without depleting populations or losing wild species in other areas [91]. In places where legal user rights over wildlife have been granted to either private land owners or communities in parts of southern Africa, wildlife populations have generally increased dramatically [92,93]. However, such approaches can also impart problems if poorly managed, as in cases of evolutionary consequences driven by selective human harvesting for particular phenotypic traits [94], exacerbation of exploitation of rare species due to higher prices [95], fragmentation of populations with fencing [65], overstocking of large wild herbivores [91], increased intolerance towards carnivores [91] and violations to animal welfare [96].

### 5.3. Alternative foods

Providing alternative foods or incomes for communities who depend unsustainably on wildlife hunting is of key importance as research shows that subsistence hunting is a ‘poor person's game’, often conducted when the hunter has no viable alternatives [47]. Attempts to provide subsistence hunters access to livestock husbandry projects have rarely been successful in reducing hunting offtakes [97]. One problem is that hunting returns tend to be highly biased, with a few individuals responsible for most of the harvest. The most successful hunters are the hardest to engage in alternatives, having the most to lose, and can maintain the community's unsustainable offtakes even if other hunters move to other activities [78]. Shifting to alternative prey species that are more resilient to hunting is also an option, particularly in post-depletion scenarios where the most susceptible target species are no longer available in exploitable densities [41,98].

Although justifications for wild meat harvest in terms of food for impoverished communities must be weighed seriously, it is critical to acknowledge that the terms ‘protein’ and ‘meat’ are not synonymous. Historically, many cultures from around the world obtained the vast majority of protein calories from plants and not animals—either wild or domestic [99,100]. Consumption of high-protein plant foods such as soy, pulses, cereals and tubers can satisfy protein requirements that are associated with fewer environmental impacts than livestock or wild meat, while yielding significant human health benefits [100–102]. In some areas, using traditionally grown protein-rich plant foods rather than wild meat as a primary protein source for humans could help mitigate the wildlife crisis if cropland is available or plant-based food products can be imported. Additionally, plant-based meat substitutes (meat analogues), driven by technological advances, are now well established in developed countries, but probably not yet commonly available in many developing countries. Furthermore, novel sources of protein and miconutrient-rich plant foods such as microalgae or seaweed could also be environmentally sustainable and useful in overcoming unsustainable hunting, hunger and malnutrition [103]. Compared with the production of animal protein from large-bodied livestock which is fraught with many downfalls and negative environmental impacts [13,14], invertebrates, which require little land and tend to have lower environmental impacts, and more regular returns, may provide a critical alternative form of protein-rich food [103]. Integrated agriculture–aquaculture systems, historically common especially in Southeast Asia can have high food efficiencies and diverse sources of aquatic protein production [104]. Ultimately, reducing global consumption of meat is a key step necessary to reduce both negative impacts of human hunting on wild animals and environmental problems associated with livestock production [96]. In addition to reducing meat consumption, improving cropping efficiencies, curtailing tropical forest conversion to agriculture and curbing food wastes would sustainably help improve food security while protecting the environment [105–107].

### 5.4. Education and family planning

**Figure 5.**
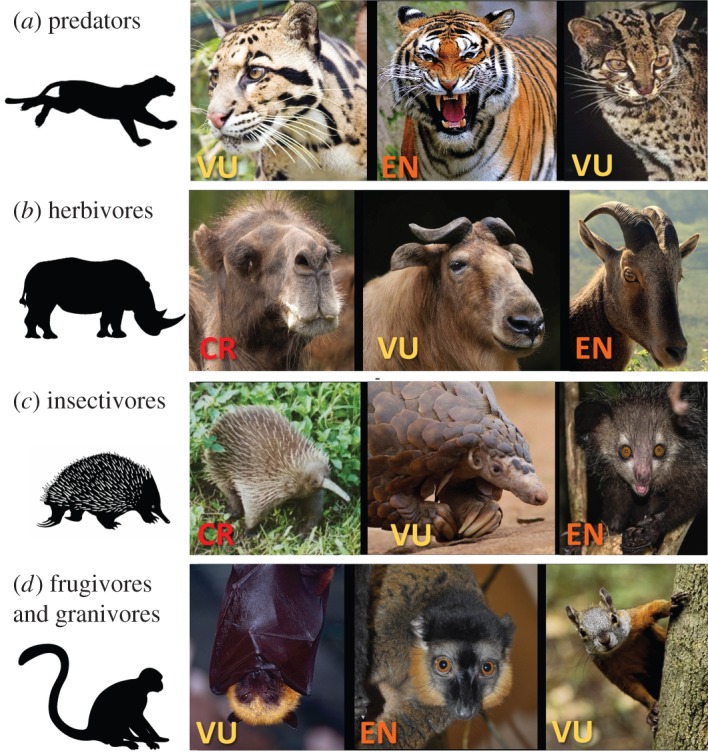
Mammal species threatened by hunting span a range of taxonomic and trophic groups, and perform a wide range of functional roles, ranging from seed dispersal to pest control to ecosystem engineering and regulation. Endangerment classification for each species noted on the image. Status categories are vulnerable (VU), endangered (EN) and critically endangered (CR). See the electronic supplementary material for photo credits. See electronic supplementary material, table S1 for population trends and endangerment category definition and trends. (*a*) Predators, left to right: clouded leopard (*Neofelis nebulosa*), tiger (*Panthera tigris*), marbled cat (*Pardofelis marmorata*). (*b*) Herbivores, left to right: Bactrian camel (*Camelus ferus*), takin (*Camelus ferus*)*,* Nilgiri tahr (*Nilgiritragus hylocrius*). (*c*) Insectivores, left to right: long-beaked echidna (*Zaglossus bruijnii*), giant ground pangolin (*Smutsia gigantea*), aye aye (*Daubentonia madagascariensis*). (*d*) Frugivores & granivores, left to right: Madagascan fruit bat (*Pterofus rufus*), collared brown lemur (*Eulemur collaris*) and Sulawesi giant squirrel (*Rubrisciurus rubriventer*).

Environmental education campaigns can reduce demand for highly valuable species. For example, shark fin commerce has apparently declined substantially subsequent to effective social media campaigns featuring appeals by Chinese basketball celebrity Yao Ming [108]. For charismatic or iconic wildlife species threatened by commercial hunting (figure 5), well-organized pleas by celebrities could be helpful for reducing demand, but this is unlikely to be effective for species relied upon for food security. However, it is notable that in many instances, particularly in urban areas, bushmeat consumption is positively correlated with wealth [109]. In this case, bushmeat is consumed in large part as a cultural legacy, being a delicacy or sign of affluence, not as a survival necessity, and thus this behaviour may be more vulnerable to social pressure. Culture is a strong force in shaping human behaviours and preferences, but is slow to change and usually unreceptive to formal education programmes unless there are financial incentives. Even in the face of the immediate danger of emerging diseases, hunters and wild meat consumers have been resistant to changing their cultural routines [110]. However, cultural norms can change rapidly given strong pressure, as was the case for reducing the demand for shark fins.

In regions with rapidly growing human populations, programmes helping to lower human birth rates are needed, especially those in rural areas that enhance education opportunities for young women. For example, this need is obvious in Madagascar where the human population has almost doubled in the last 20 years and many of the rural people, living under conditions of poverty, are severely impacting the remaining forest habitat fragments while overhunting and eating numerous threatened lemur (*Lemuroidea*) species [111]. Likewise, there is an unmet need for family planning in sub-Saharan Africa where 25% of women ages 15–49 reported that they wanted to avoid or delay pregnancy but had not used contraceptives [112]. Furthermore, at the global scale, some 225 million women want to avoid pregnancies but do not have an effective contraceptive method [113]. Providing adequate contraception in developing countries as a top priority would cost only an additional $5.3 billion annually [113]. In addition to reducing pressure on the world's hunted species, lowering human fertility rates would yield other public health, environmental and climate change mitigation co-benefits [114].

### 5.5. International policy

Although overhunting of wild meat is primarily a problem in developing countries, wealthier nations exacerbate or even drive the problem by inflating demand and prices for meat, trophy, medicinal and ornamental products. For example, there is massive demand for various wildlife products in China and several other Asian nations for both traditional medicine and for food, resulting in significant illegal hunting of a number of species, including iconic animals such as elephants, rhinos and tigers. More than 260 tons of wild meat per year were estimated to be smuggled in personal baggage into just one European airport (Paris Roissy-Charles de Gaulle) in 2008 [115]. Furthermore, multinational companies extracting natural resources in developing countries have a responsibility to ensure that increasing access to wild lands and wild animals does not introduce new non-subsistence hunting impacts. Overfishing by commercial fishing activities affect not only marine ecosystems but may also inadvertently drive hunting on land, because fish represent a dietary substitute for bushmeat [116]. The explosive increase of both non-domestic (e.g. heavily subsidized European Union) fishing and its harvest in African waters appears to be an unexpected driver of the wild meat crisis by forcing local populations to depend on terrestrial bushmeat instead of historically sustainable marine resources. The good governance of forums and multilateral agreements such as the Convention on Biodiversity, the London Declaration on Illegal Wildlife Trade, and the Convention on International Trade in Endangered Species (CITES) will be important in determining our ability to stem global trade and to share the financial and governmental burden of responsibility between the hunting nations and the demanding nations. Further, the United Nations Reducing Emissions from Deforestation and Degradation (REDD+) programme, which strives to attribute financial value to the carbon stored in forests, also offers potential to serve as a tool for biodiversity conservation. Incentives to reduce emissions are intended to be implemented in ways that are consistent with the conservation of biological diversity [117]. Though there are currently no explicit bushmeat-related components to REDD+ [118], this mechanism nevertheless offers *potential* to reduce bushmeat exploitation both indirectly, via reducing the prevalence of logging roads (and hunter access), and directly, via formalized limitations to hunting.

## 6. Concluding remarks

Our analysis illustrates how human hunting for consumption of food and medicine is contributing to extinction risk for many terrestrial mammal species. The complexity of managing the wild meat trade lies in the fact that while rural dependency on wild meat is often a necessity under current conditions [77], the commodity is frequently a luxury in urban areas [119] and these two scenarios require very different management tools. In addition, the demand for wild meat is often exacerbated by simultaneous demand for the same species for medicinal, ornamental or trophy use, particularly in Asia [120], and so the same hunter or trader may be responding to multiple drivers. Our options for changing human demand for threatened wildlife must be encapsulated in internationally cohesive, pragmatic policies and action plans such as those proposed in the recent London Declaration (2014), which was signed by 46 countries. These call for a broad array of actions to stem illegal wildlife trade including eradicating markets for illegal wildlife products, ensuring effective legal frameworks and deterrents, strengthening law enforcement and promoting sustainable livelihoods and development. Furthermore, developed countries committed to providing significant funding for biodiversity in the tropics at the Rio 1992 Earth Summit, but have not fulfilled those promises. If such funding were to become available, developed countries could play a significant role in helping developing countries protect their wildlife from illegal hunting for meat and other body parts. All of these actions, along with several others are embedded in the five conservation actions that we describe above for reducing both wildlife mortality and demand for wild meat and products.

Wild meat provides a primary food source for many millions of people throughout the developing world, especially where other food options are not readily available [39]. Unsustainable hunting has now metamorphosed into a global hunting crisis taking the form of a serious threat to the food security of many people as well as the immediate survival of hundreds of mammal species, other wildlife and altered ecological cascades rippling through ecosystems. Averting this global crisis requires bold, ambitious and prompt actions involving many mainstream economic and policy sectors. New actions are needed to address all aspects of the crisis. Approaches that benefit both local people and wildlife will be required to avoid a future of hungry desperate people inhabiting ‘empty landscapes’ across much of the planet Earth.

## Supplementary Material

Supplement to “Bushmeat hunting and extinction risk to the world's mammals”

## Data accessibility

All data needed to evaluate the conclusions in the paper are present in the paper and the electronic supplementary material. Additional data related to this paper can be downloaded from the IUCN Red List website at http://www.iucnredlist.org/.
