# Supplementary material for: Bushmeat hunting and extinction risk to the world's mammals
Source: R Soc Open Sci. 2016 Oct 19;3(10):160498. doi: 10.1098/rsos.160498 (PMC5098989; doi:10.1098/rsos.160498)
Supplement: Supplement to “Bushmeat hunting and extinction risk to the world's mammals” [file rsos160498supp1.pdf]

Supplementary Material for

Bushmeat hunting and extinction risk to the world's mammals

William J. Ripple, Katharine Abernethy, Matthew G. Betts, Guillaume Chapron, Rodolfo Dirzo, Mauro Galetti, Taal Levi, Peter A. Lindsey, David W. Macdonald, Brian Machovina, Thomas M. Newsome, Carlos A. Peres, Arian D. Wallach, Christopher Wolf, Hillary Young

*Corresponding author. E-mail: bill.ripple@oregonstate.edu

Published 19 October 2016.DOI: 10.1098/rsos.160498

This PDF file includes:
Methods
Scientific names and photo credits for Figure 5
Figs. S1 to S8
Tables S1 to S3

Online Supplement

Methods

We used the International Union for Conservation of Nature (IUCN) Red List to identify mammals threatened by hunting (1). We started with the 5,508 mammals listed by the IUCN (including some data deficient species). All of these species were last assessed in 2008 or later. We then excluded the 127 marine mammals other than otters and the polar bear (*Ursus maritimus*), giving a list of 5,374 species. We then restricted the list to species that have endangerment statuses LC (least concern), NT (near threatened), VU (vulnerable), EN (endangered), or CR (critically endangered). This resulted in a list of 4,556 terrestrial mammals, which we refer to as the assessed terrestrial mammals.

Of these 4,556 mammals, we examined the 1,169 species that are classified as threatened with extinction (VU, EN, or CR). In addition to their endangerment statuses, the IUCN lists threats faced by each species. There are 12 categories: "Residential & commercial development," "Agriculture & aquaculture," "Energy production & mining," and so on. We looked at the species listed as facing threat 5.1.1: "Biological resource use" > "Hunting & collecting terrestrial animals" > "Intentional use (species being assessed is the target)." The IUCN lists examples of this threat as "bushmeat hunting, trophy hunting, beaver trapping, butterfly collecting, honey or bird nest hunting, etc." Of the 1,169 threatened terrestrial mammals, we restricted our list to the 496 species facing threat 5.1.1 (intentional use).

We then scrutinized each of the 496 species individually to find species threatened by hunting. Specifically, we looked at the "Major Threat(s)" section of the fact sheet for each species. If one of the major threats for a species was hunting (for meat or body parts), then we considered that species threatened by hunting. We found 301 such species. In addition, we recorded other major threats each species faced. The threats we considered were: "meat hunting," "body parts hunting" (specific body parts noted whenever possible), "medicine," "ornaments," "live trade," "pets," "pest/eradication," "habitat loss," "deforestation," "agriculture," "human encroachment," "livestock competition," "disease," "civil unrest," "hybridize with livestock," "predators," "pollution," "drought," "prey base," "hybridize in genus," "cyclones/typhoons," and "fires."

Species Body Masses

We obtained species body mass data primarily from the PanTHERIA mammal species database (1). Species masses are defined there as: "Mass of adult (or age unspecified) live or freshly-killed specimens (excluding pregnant females) using captive, wild, provisioned, or unspecified populations; male, female, or sex unspecified individuals; primary, secondary, or extrapolated sources; all measures of central tendency; in all localities" (1). This database included only 182 (60.5%) of the 301 species threatened by hunting on our list. We therefore obtained 34 additional species masses from (2) and 17 from (3), giving a total of 233 species masses (77.4% of all 301 species).

We linked research effort (in terms of numbers of published articles) with body mass using negative binomial regression. Only species with known masses were used for the regression. We used body mass (log transformed) as the explanatory variable. Negative binomial regression was used instead of Poisson regression in order to allow for over dispersion. We found a significant ($p < 0.001$) positive relationship between mass and research effort, with a doubling in species mass associated with a 33% increase in mean number of published articles. A 95% confidence interval for this increase was (22%, 46%).

Research Effort

We estimated the relative research effort allocated to each of the 301 species threatened by hunting using Thomson Reuters' Web of Science. We searched all available Web of Science databases for publications with the topic (title, abstract, author keywords, or Web of Science keywords) matching each species' scientific name or any of the taxonomic synonyms listed on the species' IUCN Red List fact sheet page. We then recorded the number of publications, restricting our search to the time range 1965-2016.

Species Richness Index Mapping

We mapped species richness using IUCN Red List range maps (5). For each species with a range map, we restricted its range to regions where it was classified as "extant" or "probably extant." To better highlight species with restricted ranges, we converted the range maps to a hexagonal grid with hexagon size ~70,000 km^2 (6).We considered a species present in a hexagon if any part of its range overlapped that hexagon. We then mapped the numbers of mammals, mammals threatened by hunting, and mammals threatened by each major hunting and habitat loss type by

counting the number of each group of species in each hexagon. We used the same approach to find the median number of published articles for the species threatened by hunting in each hexagon.

Change in Endangerment

We obtained corrected 1996 species endangerment statuses from IUCN per Michael Hoffmann. For these data, Hoffmann et al. determined corrected endangerment statuses by making corrections to reflect updated taxonomic information, fix incorrect assessments, and account for changes to Red List methodology (7). When analyzing change in endangerment, we compare 1996 status with the most recent status available for each species. The year of most recent assessment varied from species to species, but was always 2008 or later.

Country/Regional Analysis

For each of the 4,556 terrestrial mammal species (including the 301 species threatened by hunting), we listed the countries (if any) where it was classified as native (and present) according to its IUCN Red List species fact sheet. We then used this information to count the number of species threatened by hunting in each country.

In addition, we aggregated countries into regions to find the percentage of threatened and threatened by hunting species in each region. The regional grouping we used was based on the one given in (8). For "Asia," we excluded South-Eastern Asia, which we treated separately. We grouped Central America, South America, and the Caribbean together as "Latin America." We treated Russia as part of Asia, rather than Europe.

We also considered the developed and developing worlds as another regional split. We treated North America, Europe, Russia, Japan, Australia, and New Zealand as the developed world (8). Of the 301 species in our analysis, four (kouprey, Telefomin cuscus, Wondiwoi tree-kangaroo, and little earth hutia) are classified as "possibly extinct" everywhere. For this part of our analysis, we treated each of those species as being found exclusively in the developing world since in each case, the countries the species was listed as "possibly extinct" from belong to the developing world.

Protected Areas Analysis

We used the World Database on Protected Areas (WDPA) to assess overlap between the 301 hunted mammal species' ranges and protected areas (9). This database contains spatial data for 217,300 protected areas (91% polygons and 9% points). We used only the polygon data, rather than buffering the point locations, in order to avoid overestimating overlap. Because of this, our overlap estimates may be conservative for some species. For our analysis, we only used strictly protected areas, corresponding to IUCN management categories Ia (Strict nature reserve), Ib (Wilderness area), II (National Park), or III (Natural Monument or feature), as these are generally considered benchmarks for biodiversity conservation (9,10). For the 301 hunted mammal species' ranges, we used IUCN Red List range maps and only considered regions where the species were classified as extant or probably extant. We calculated species' range areas and then clipped species' ranges to protected areas to determine overlap areas. All areas were calculated using the Mollweide equal-area projection.

Scientific names and photo credits for Figure 5

Predators

1) Clouded leopard (*Neofelis nebulosa*). Photo by Susan Shepard, licensed under CC BY 2.0 via Flickr.
2) Tiger (*Panthera tigris).* Photo by Claudio Gennari licensed under CC BY 2.0 via Flickr.
3) Marbled cat (*Pardofelis marmorata*) Photo by Madeleine Deaton, licensed under CC BY 2.0 via Flickr.

Herbivores

1) Bactrian camel (*Camelus ferus*). Photo by Weisserstler, licensed under CC BY 2.0 via Flickr.
2) Takin (*Budorcas taxicolor*). Photo by Su Neko, licensed under CC BY 2.0 via Flickr.
3) Nilgiri Tahr (*Nilgiritragus hylocrius*). Photo by Sankara Subramanian licensed under CC BY 2.0 via Flickr.

Insectivores

1) Long beaked echidna (*Zaglossus bruijnii*). GNU Free documentation license.
2) Giant ground pangolin (*Smutsia gigantean*). Photo by David Brossard, licensed under CC BY-SA 2.0 via Flickr.
3) Aye-aye (*Daubentonia madagascariensis*). Photo by Nomis-Simon licensed under CC BY 2.0 via Flickr.

Frugivores & Granivores

1) Madagascan fruit bat (*Pterofus rufus*). Photo by Bernard Dupont, licensed under CC BY 2.0 via Flickr.
2) Collared brown lemur (*Eulemur collaris*). Photo by Elias Neideck licensed under CC BY SA 3.0 via Wikimedia

3) Sulawesi giant squirrel (*Rubrisciurus rubriventer*). Photo by Thomas Simonsen, reproduced with permission.

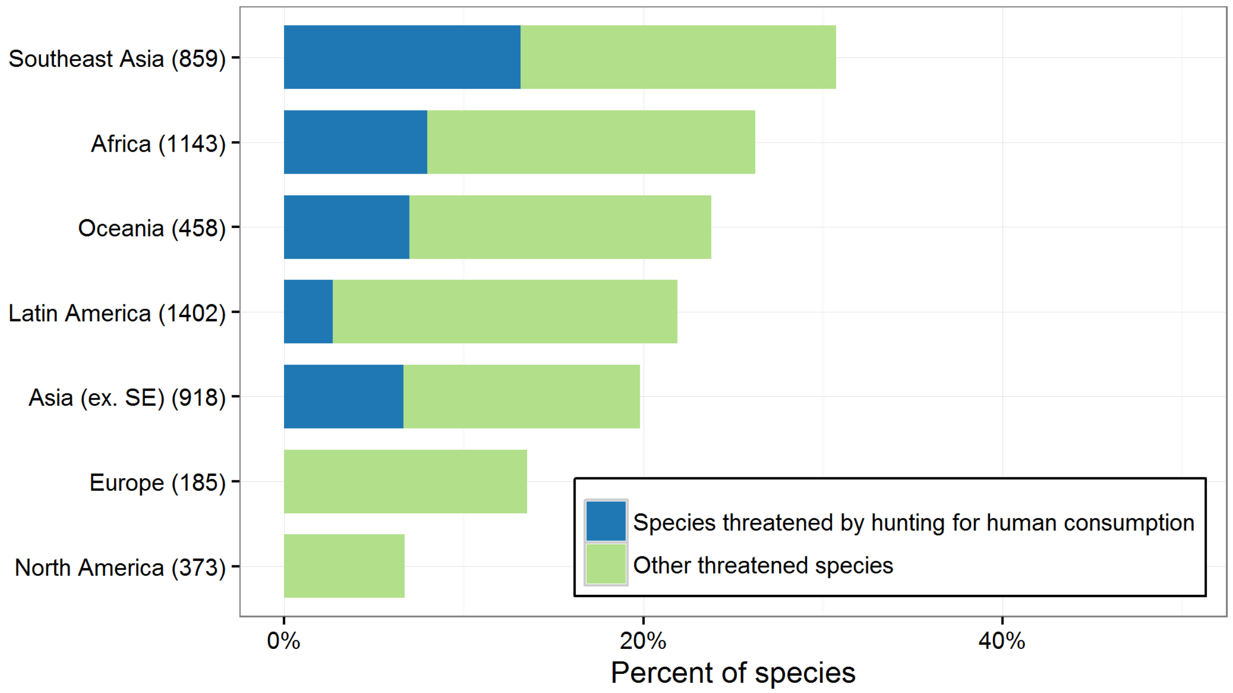

Figure S1. Percentages of mammal species threatened by hunting for human consumption in each world region along with the percentages of other threatened species. The values on the x-axis refer to the percentage of species out of all mammal species found in each region. The bars are sorted by length, which indicates the total percentage of species that was threatened in each region. The numbers in parentheses represent the total number of (mammal) species found in each region.

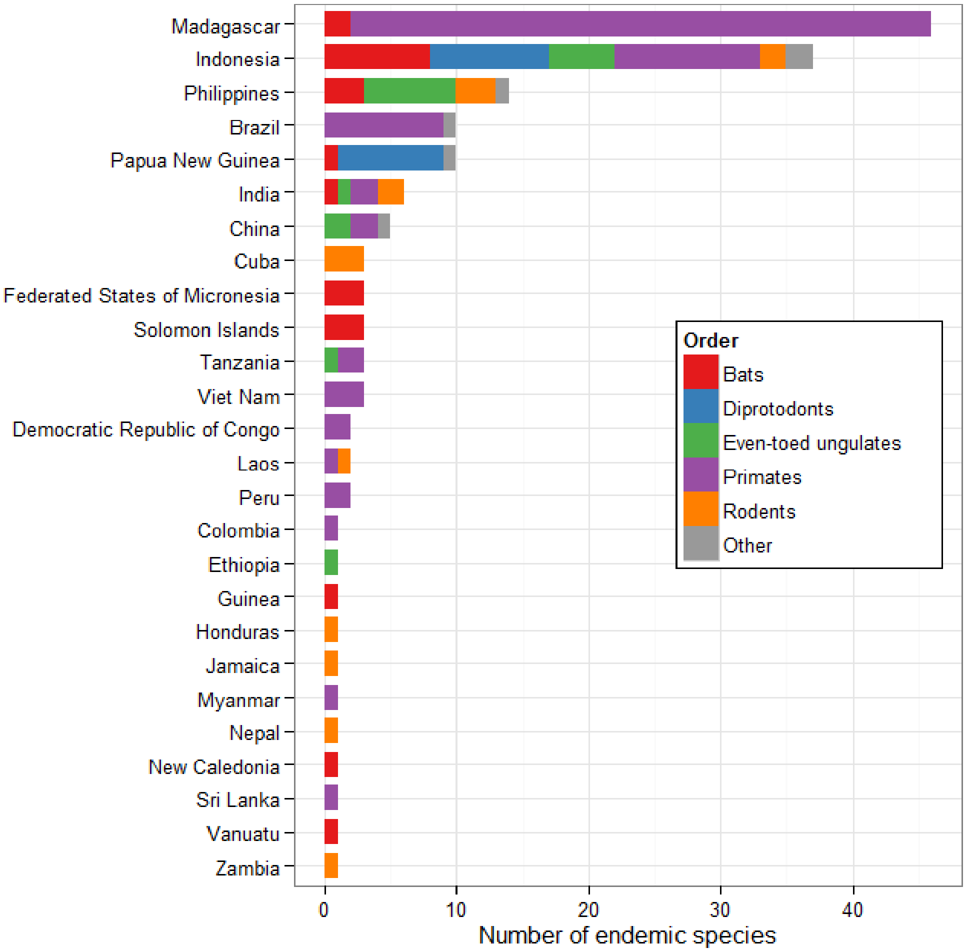

Figure S2. Countries with at least one endemic species threatened by hunting. We considered endemic species to be those native and present in only one country. The colors show mammalian orders. There are a total of 160 endemic mammals threatened by hunting.

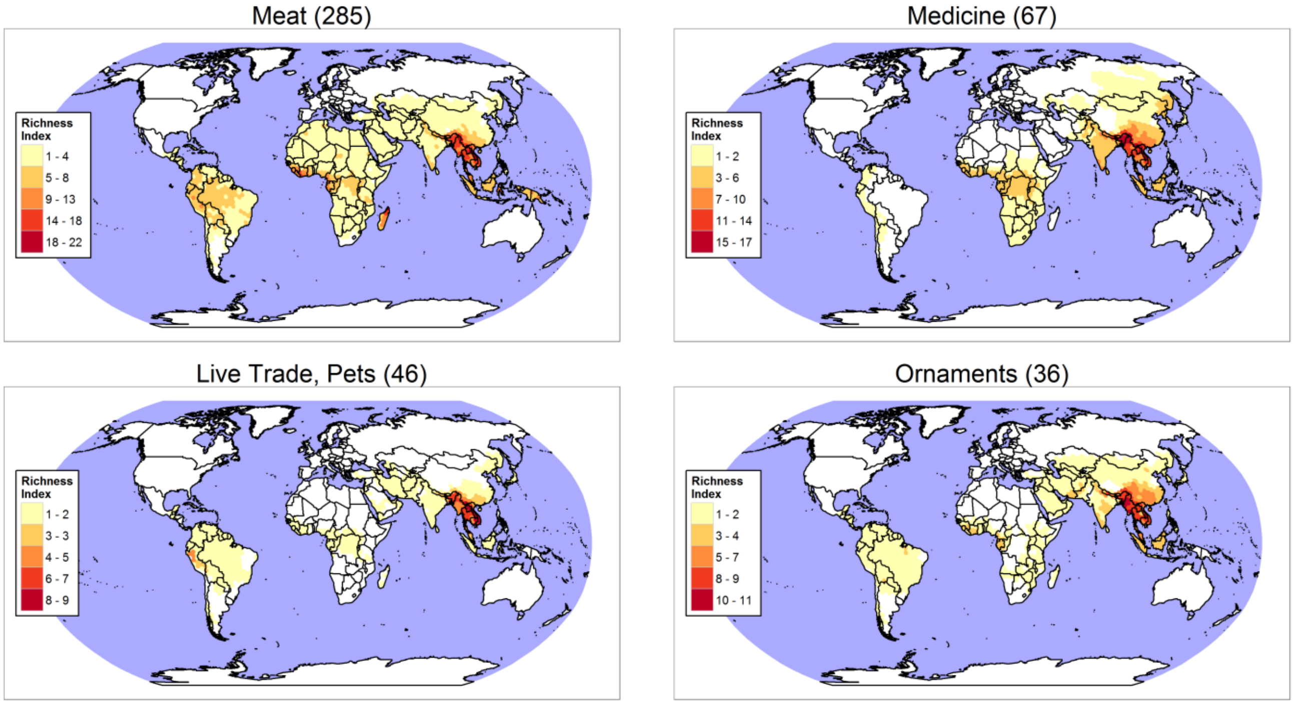

Figure S3. Top four reasons why humans hunt mammals. The numbers of species facing each threat is shown in parentheses. Meat consumption (top left) was the most common reason for hunting.

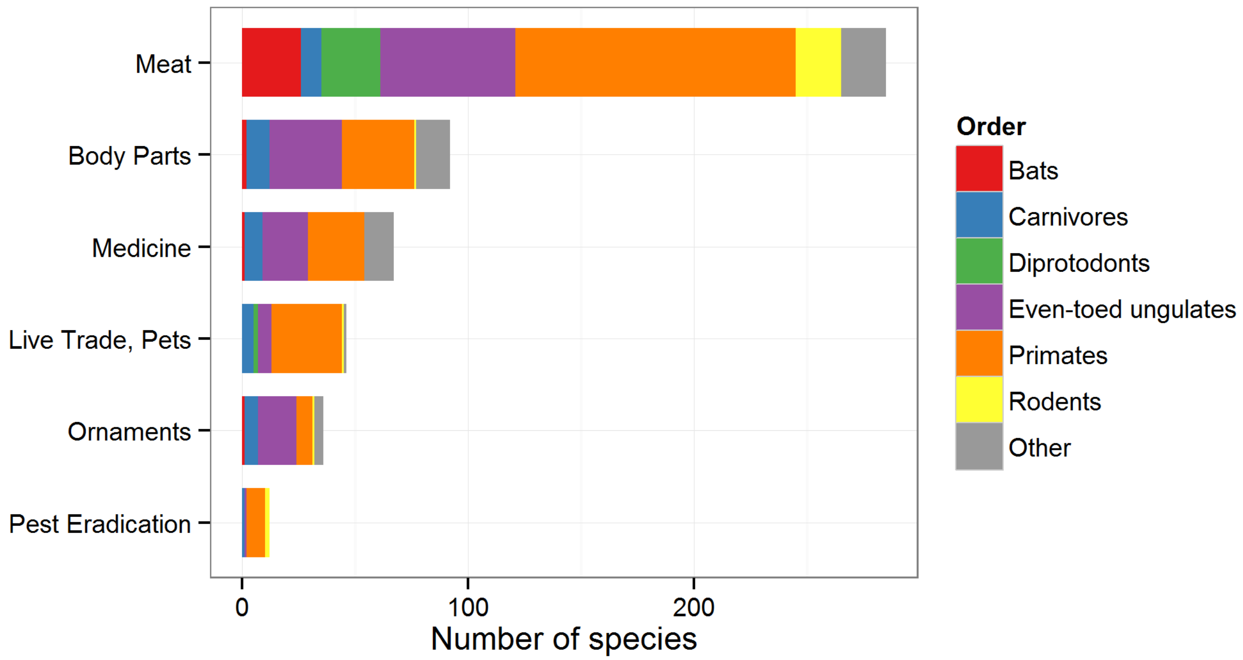

Figure S4. Reasons why humans hunt mammals. Only orders with at least 10 threatened hunted species are labeled.

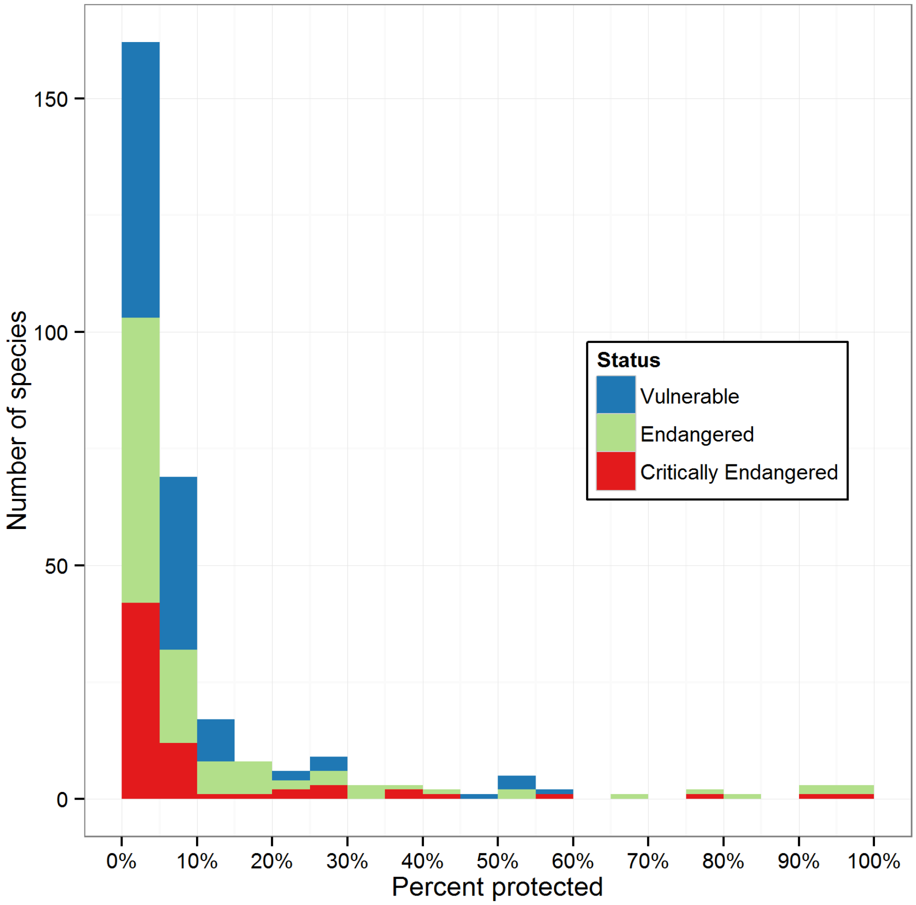

Figure S5. Percentages of species ranges that overlap protected areas. For example, 162 species have less than 5% of their ranges within protected areas. Only results for the 298 species that are extant or probably extant are shown.

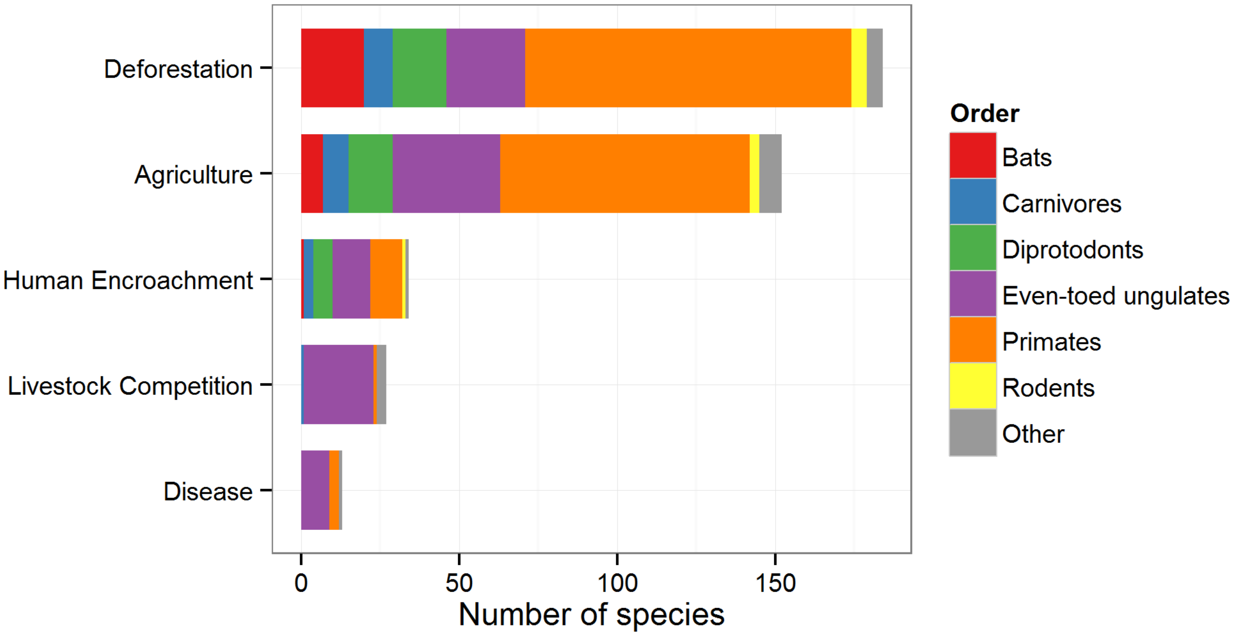

Figure S6. Compounding threats on heavily hunted wildlife. Only categories and orders with at least 10 species within a threat type are shown.

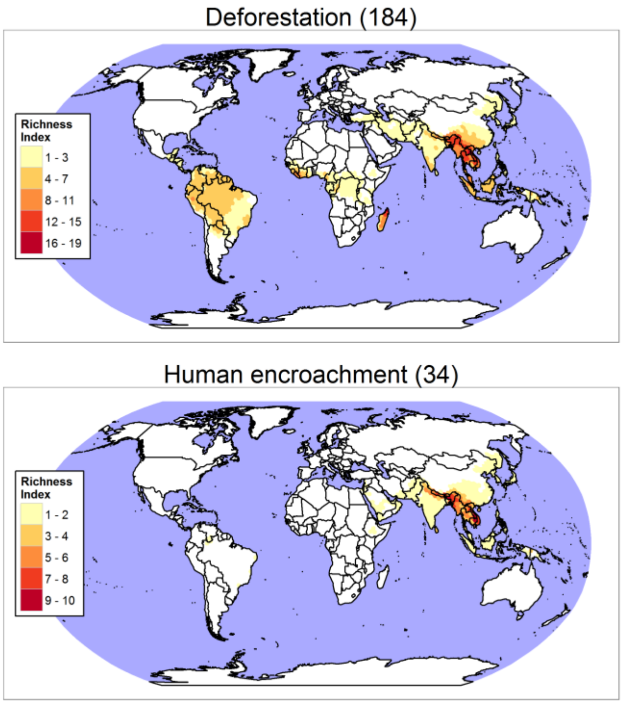

Figure S7. Habitat-related coexisting threats faced by mammal species threatened by hunting. The numbers of species facing each threat is shown in parentheses. Unlike the other threats, competition with livestock was relatively common in North Africa, where productivity is lower.

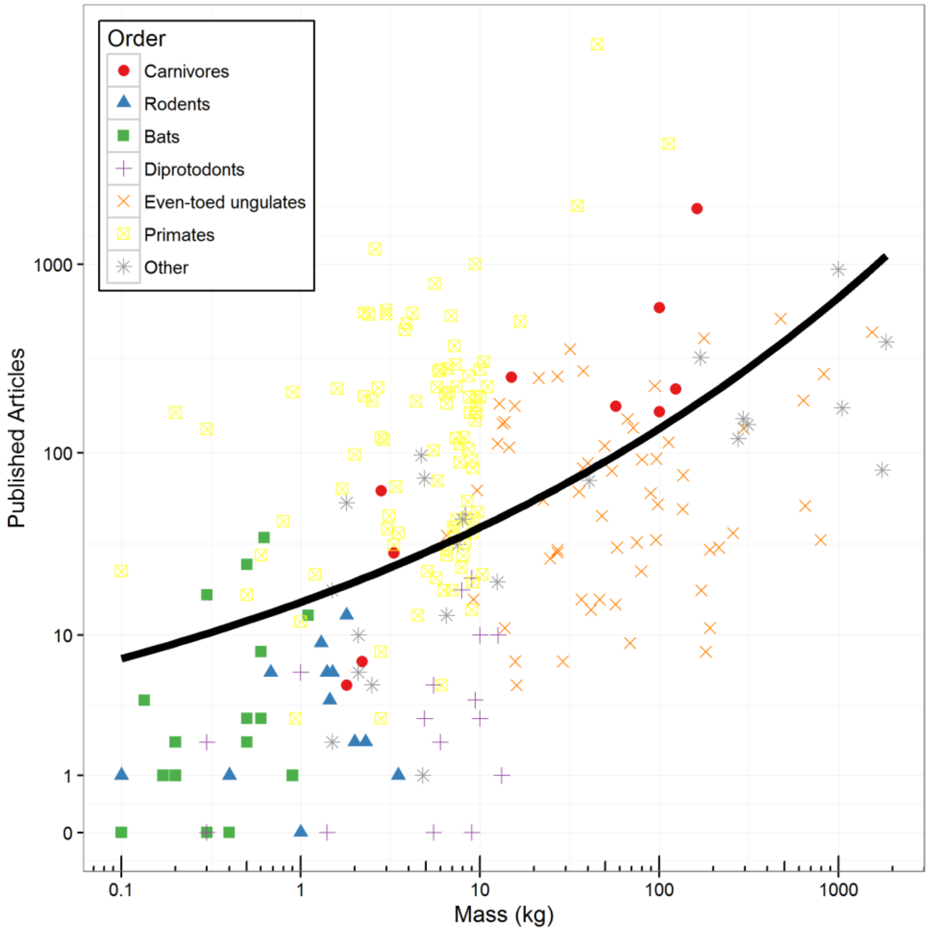

Figure S8. Published articles (according to Thomson Reuters' Web of Science) versus body mass for 233 threatened hunted species. The black line shows a negative binomial regression fit for the mean number of articles. We found a significant positive relationship between log species mass and research effort ($p < 0.001$) with a doubling in species body mass associated with a 36% increase in the mean number of published articles.

Table S1. Basic information on the 301 heavily hunted and threatened mammal species including scientific names, common names, endangerment status in 1996, most recent endangerment status as of 2014 (last two digits of status years when other than 2008 are shown in parentheses), population trends (decreasing, stable, increasing, or unknown), and body masses. The year of most recent assessment varied from species to species, but was always 2008 or later. Note that a few species where not assessed in 1996 and for some species body masses were unknown to us. Status categories are Least Concern (LC), Near Threatened (NT), Vulnerable (VU), Endangered (EN), Critically Endangered (CR), and Data Deficient (DD).

Family	Scientific Name	Common Name	Status 96	Status	Trend	Mass (kg)
**Carnivora (carnivores)**						
Felidae	Panthera tigris	Tiger	EN	EN (11)	Dec	161.9
Ursidae	Tremarctos ornatus	Andean Bear	VU	VU	Dec	123.2
Ursidae	Melursus ursinus	Sloth Bear	VU	VU	Dec	100
Ursidae	Ursus thibetanus	Asiatic Black Bear	VU	VU	Dec	99.7
Ursidae	Helarctos malayanus	Malayan Sun Bear	VU	VU	Dec	57.1
Felidae	Neofelis nebulosa	Clouded Leopard	VU	VU	Dec	14.9
Viverridae	Chrotogale owstoni	Owston's Banded Palm Civet	VU	VU	Dec	3.3
Felidae	Pardofelis marmorata	Marbled Cat	VU	VU	Dec	2.8
Viverridae	Genetta johnstoni	Johnston's Genet	VU	VU	Dec	2.2
Herpestidae	Liberiictis kuhni	Liberian Mongoose	VU	VU (12)	Dec	1.8
Felidae	Leopardus jacobita	Andean Cat	EN	EN	Dec	
Viverridae	Viverra megaspila	Large-spotted Civet	NT	VU	Dec	
**Cetartiodactyla (even-toed ungulates)**						
Hippopotamidae	Hippopotamus amphibius	Common Hippopotamus	VU	VU	Dec	1536.3
Bovidae	Bos gaurus	Gaur	VU	VU	Dec	825
Bovidae	Bos sauveli	Grey Ox	CR(PE)	CR	Unk	791.3
Bovidae	Bos mutus	Wild Yak	VU	VU	Dec	650
Bovidae	Bos javanicus	Banteng	EN	EN	Dec	636
Camelidae	Camelus ferus	Bactrian Camel	EN	CR	Dec	475
Bovidae	Budorcas taxicolor	Takin	VU	VU	Dec	294.5
Bovidae	Bubalus depressicornis	Anoa	EN	EN	Dec	257
Bovidae	Tragelaphus buxtoni	Mountain Nyala	EN	EN	Dec	215
Suidae	Sus cebifrons	Visayan Warty Pig	CR	CR	Dec	190.8
Suidae	Sus philippensis	Philippine Warty Pig	VU	VU	Dec	190.8
Bovidae	Bubalus quarlesi	Mountain Anoa	EN	EN	Dec	181.7
Cervidae	Rusa unicolor	Sambar	VU	VU	Dec	177.5
Cervidae	Rucervus duvaucelii	Barasingha	VU	VU (13)	Dec	171.2
Suidae	Sus barbatus	Bearded Pig	NT	VU	Dec	135.8
Cervidae	Cervus albirostris	Thorold's Deer		VU (14)	Unk	135
Cervidae	Blastocerus dichotomus	Marsh Deer	VU	VU	Dec	112.5
Bovidae	Pseudoryx nghetinhensis	saola	EN	CR	Dec	97.8

Family	Scientific Name	Common Name	Status 96	Status	Trend	Mass (kg)
Bovidae	Addax nasomaculatus	Addax	EN	CR	Dec	96.1
Cervidae	Rucervus eldii	Brow-antlered Deer	EN	EN	Dec	95.5
Bovidae	Ammotragus lervia	Aoudad	VU	VU	Dec	94.2
Suidae	Sus verrucosus	Javan Pig	EN	EN	Dec	89.4
Suidae	Babyrousa celebensis	Sulawesi Babirusa	VU	VU	Dec	80
Bovidae	Beatragus hunteri	Hirola	CR	CR	Dec	79.1
Bovidae	Nilgiritragus hylocrius	Nilgiri Tahr	EN	EN	Dec	75
Bovidae	Nanger dama	Addra Gazelle	EN	CR	Dec	71.4
Bovidae	Cephalophus jentinki	Jentink's Duiker	EN	EN	Dec	68.5
Cervidae	Rusa timorensis	Javan Deer	NT	VU	Dec	66.4
Bovidae	Capra caucasica	West Caucasian Tur	VU	EN	Dec	58.1
Bovidae	Cephalophus spadix	Abbott's Duiker	VU	EN	Dec	56.9
Bovidae	Capra falconeri	Markhor	EN	EN	Dec	54.3
Cervidae	Rusa marianna	Philippine Brown Deer	VU	VU	Dec	49.5
Bovidae	Capra nubiana	Nubian Ibex	VU	VU	Dec	47.8
Cervidae	Rusa alfredi	Phillipine Spotted Deer	EN	EN	Dec	46.5
Bovidae	Nanger soemmerringii	Soemmerring's Gazelle	VU	VU	Dec	41.6
Cervidae	Axis calamianensis	Calamanian Deer	EN	EN	Dec	39.8
Bovidae	Saiga tatarica	Mongolian Saiga	VU	CR	Dec	37.7
Cervidae	Axis porcinus	Hog Deer	EN	EN (12)	Dec	37.4
Cervidae	Muntiacus vuquangensis	Giant Muntjac	EN	EN	Dec	36.7
Tayassuidae	Catagonus wagneri	Chacoan Peccary	EN	EN (14)	Dec	35.6
Tayassuidae	Tayassu pecari	White-lipped Peccary	LC	VU (13)	Dec	31.8
Bovidae	Naemorhedus baileyi	Red Goral	VU	VU	Dec	28.9
Bovidae	Naemorhedus caudatus	Chinese Goral	VU	VU	Dec	27
Bovidae	Eudorcas rufifrons	Red-fronted Gazelle	VU	VU	Dec	27
Bovidae	Gazella subgutturosa	Goitered Gazelle	NT	VU	Dec	27
Bovidae	Gazella leptoceros	Rhim	EN	EN	Dec	24.6
Bovidae	Gazella cuvieri	Cuvier's Gazelle	EN	EN	Unk	22.5
Bovidae	Gazella gazella	Idmi	VU	VU	Dec	21.3
Moschidae	Moschus anhuiensis	Anhui Musk Deer	EN	EN	Dec	16
Bovidae	Cephalophus zebra	Banded Duiker	VU	VU	Dec	15.7
Bovidae	Gazella dorcas	Dorcas Gazelle	NT	VU	Dec	15.6
Moschidae	Moschus leucogaster	Himalayan Muskdeer	EN	EN	Dec	14.5
Moschidae	Moschus chrysogaster	Alpine Musk Deer	EN	EN	Dec	13.7
Moschidae	Moschus fuscus	Black Musk Deer	EN	EN	Dec	13.7
Moschidae	Moschus moschiferus	Siberian Musk Deer	VU	VU	Dec	13.3
Cervidae	Hydropotes inermis	Chinese Water Deer	NT	VU	Dec	12.8
Moschidae	Moschus berezovskii	Chinese Forest Musk Deer	EN	EN	Dec	12.5
Cervidae	Pudu puda	Chilean Pudu	VU	VU	Dec	9.6
Bovidae	Cephalophus adersi	Aders' Duiker	EN	CR	Dec	9.2
Tragulidae	Tragulus nigricans	Balabac Chevrotain	VU	EN	Dec	6.5
Bovidae	Capra aegagrus	Bezoar	NT	VU	Dec	

Family	Scientific Name	Common Name	Status 96	Status	Trend	Mass (kg)
Moschidae	Moschus cupreus	Kashmir Muskdeer	EN	EN	Dec	
Bovidae	Naemorhedus griseus	Chinese Goral	VU	VU	Dec	
Bovidae	Ovis orientalis	Cyprian Wild Sheep	VU	VU	Dec	
Suidae	Sus ahoenobarbus	Palawan Bearded Pig	VU	VU	Dec	
		**Chiroptera (bats)**				
Pteropodidae	Acerodon jubatus	Golden-capped Fruit Bat	EN	EN	Dec	1.1
Pteropodidae	Pteropus melanopogon	Black-bearded Flying Fox	VU	EN	Dec	0.9
Pteropodidae	Aproteles bulmerae	Bulmer's Fruit Bat	CR	CR	Dec	0.6
Pteropodidae	Pteralopex anceps	Bougainville Monkey-faced Bat	EN	EN	Dec	0.6
Pteropodidae	Pteropus rufus	Madagascan Flying Fox	VU	VU	Dec	0.6
Pteropodidae	Pteralopex atrata	Cusp-toothed Flying Fox	EN	EN	Dec	0.5
Pteropodidae	Acerodon mackloti	Sunda Flying-fox	VU	VU	Dec	0.5
Pteropodidae	Pteropus mariannus	Marianas Flying Fox	EN	EN	Dec	0.5
Pteropodidae	Pteropus pohlei	Geelvink Bay Flying Fox	EN	EN	Dec	0.4
Pteropodidae	Pteropus ornatus	Ornate Flying Fox	VU	VU	Dec	0.3
Pteropodidae	Eidolon dupreanum	Madagascan Fruit Bat	VU	VU	Dec	0.3
Pteropodidae	Pteropus ocularis	Ceram Fruit Bat	VU	VU	Dec	0.2
Pteropodidae	Pteropus fundatus	Banks Flying Fox	EN	EN	Dec	0.2
Pteropodidae	Rousettus bidens	Manado Fruit-bat	NT	VU	Dec	0.2
Pteropodidae	Harpyionycteris celebensis	Sulawesi Harpy Fruit Bat	NT	VU	Dec	0.1
Pteropodidae	Dobsonia chapmani	Negros Naked-backed Fruit Bat	CR	CR	Dec	0.1
Pteropodidae	Acerodon humilis	Talaud Acerodon	EN	EN	Dec	
Pteropodidae	Acerodon leucotis	Palawan Flying Fox	NT	VU	Dec	
Pteropodidae	Latidens salimalii	Salim Ali's Fruit Bat	EN	EN	Dec	
Pteropodidae	Pteralopex flanneryi	Greater Monkey-faced Bat	CR	CR	Dec	
Pteropodidae	Pteropus cognatus	Makira Flying Fox	EN	EN	Dec	
Pteropodidae	Pteropus insularis	Carolines Fruit Bat	CR	CR (10)	Unk	
Pteropodidae	Pteropus molossinus	Caroline Flying Fox	VU	VU	Stable	
Pteropodidae	Pteropus rennelli	Rennell Flying Fox	VU	VU	Stable	
Pteropodidae	Pteropus temminckii	Temminck's Flying Fox	VU	VU	Dec	
Pteropodidae	Pteropus yapensis	Yap Flying Fox	VU	VU	Unk	
Rhinolophidae	Rhinolophus maclaudi	Maclaud's Horseshoe Bat	EN	EN	Dec	
		**Cingulata (armadillos)**				
Dasypodidae	Priodontes maximus	Giant Armadillo	VU	VU (14)	Dec	40.6
Dasypodidae	Chaetophractus nationi	Andean Hairy Armadillo	VU	VU (14)	Dec	2.1
Dasypodidae	Tolypeutes tricinctus	Brazilian Three-banded Armadillo	VU	VU (14)	Dec	1.5
		**Diprotodontia (diprotodonts)**				
Macropodidae	Dendrolagus ursinus	Black Tree-kangaroo	VU	VU	Dec	13.2
Macropodidae	Dendrolagus inustus	Grizzled Tree Kangaroo	NT	VU	Dec	12.6
Phalangeridae	Ailurops ursinus	Bear Cuscus	VU	VU	Dec	10
Macropodidae	Dendrolagus scottae	Scott's Tree-kangaroo	EN	CR	Dec	10
Macropodidae	Dendrolagus mbaiso	Dingiso	EN	EN (10)	Dec	9.4
Macropodidae	Dendrolagus dorianus	Doria's Tree Kangaroo	VU	VU	Dec	9

Family	Scientific Name	Common Name	Status 96	Status	Trend	Mass (kg)
Macropodidae	Dendrolagus mayri	Wondiwoi Tree-kangaroo	CR(PE)	CR	Unk	9
Macropodidae	Dendrolagus notatus		EN	EN	Dec	9
Macropodidae	Dendrolagus stellarum	Seris Tree Kangaroo	NT	VU	Dec	9
Macropodidae	Dendrolagus matschiei	Huon Tree Kangaroo	EN	EN	Dec	8.3
Macropodidae	Dendrolagus goodfellowi	Goodfellow's Tree Kangaroo	EN	EN	Dec	7.9
Phalangeridae	Spilocuscus rufoniger	Black-spotted Cuscus	EN	CR	Dec	6
Macropodidae	Thylogale browni	New Guinea Pademelon	VU	VU	Dec	5.5
Macropodidae	Thylogale lanatus	Mountain Pademelon	EN	EN	Dec	5.5
Macropodidae	Dorcopsis luctuosa	Gray Dorcopsis	NT	VU	Dec	4.9
Phalangeridae	Phalanger matanim	Telefomin Cuscus	CR	CR	Unk	1.4
Pseudocheiridae	Pseudochirops coronatus	Reclusive Ringtail	NT	VU	Dec	1.4
Phalangeridae	Strigocuscus celebensis	Little Celebes Cuscus	VU	VU	Dec	1
Petauridae	Petaurus abidi	Northern Glider	CR	CR	Dec	0.3
Pseudocheiridae	Pseudochirulus schlegeli	Arfak Ringtail	VU	VU	Dec	0.3
Phalangeridae	Ailurops melanotis	Talaud Bear Cuscus	CR	CR	Dec	
Macropodidae	Dendrolagus pulcherrimus	Golden-mantled Tree Kangaroo	CR	CR	Dec	
Phalangeridae	Phalanger alexandrae	Gebe Cuscus	EN	EN	Dec	
Phalangeridae	Spilocuscus wilsoni	Biak Spotted Cuscus	EN	CR	Dec	
Macropodidae	Thylogale brunii	Dusky Pademelon	VU	VU	Dec	
Macropodidae	Thylogale calabyi	Alpine Wallaby	EN	EN	Dec	
**Lagomorpha (rabbits, hares and pikas)**						
Leporidae	Lepus hainanus	Chinese Pinyin	VU	VU	Dec	1.5
**Monotremata (platypus and echidnas)**						
Tachyglossidae	Zaglossus bruijnii	Long-beaked Echidna	CR	CR	Dec	7.5
Tachyglossidae	Zaglossus bartoni	Eastern Long-beaked Echidna	CR	CR	Dec	6.5
Tachyglossidae	Zaglossus attenboroughi	Attenborough's Echidna	CR	CR	Dec	2.5
**Peramelemorphia (bilbies and bandicoots)**						
Peramelidae	Peroryctes broadbenti	Giant Bandicoot	VU	EN	Dec	4.8
**Perissodactyla (odd-toed ungulates)**						
Rhinocerotidae	Rhinoceros unicornis	Greater One-horned Rhino	EN	VU	Inc	1843.7
Rhinocerotidae	Rhinoceros sondaicus	Javan Rhinoceros	CR	CR	Unk	1750
Rhinocerotidae	Dicerorhinus sumatrensis	Sumatran Rhinoceros	CR	CR	Dec	1046.2
Rhinocerotidae	Diceros bicornis	Black Rhinoceros	CR	CR (12)	Inc	995.9
Tapiridae	Tapirus indicus	Asian Tapir	VU	EN	Dec	311.2
Tapiridae	Tapirus bairdii	Baird's Tapir	EN	EN	Dec	293.8
Equidae	Equus africanus	African Ass	CR	CR	Dec	275
Tapiridae	Tapirus terrestris	Brazilian Tapir	NT	VU	Dec	169.5
**Pholidota (pangolins)**						
Manidae	Smutsia temminckii	Cape Pangolin	LC	VU (14)	Dec	12.5
Manidae	Manis crassicaudata	Indian Pangolin	LC	EN (14)	Dec	8
Manidae	Manis javanica	Malayan Pangolin	VU	CR (14)	Dec	4.9
Manidae	Manis pentadactyla	Chinese Pangolin	VU	CR (14)	Dec	4.7
Manidae	Manis culionensis	Philippine Pangolin	LC	EN (14)	Dec	2.1

Family	Scientific Name	Common Name	Status 96	Status	Trend	Mass (kg)
Manidae	Phataginus tricuspis	African White-bellied Pangolin	NT	VU (14)	Dec	1.8
Manidae	Smutsia gigantea	Giant Ground Pangolin	NT	VU (14)	Dec	
Manidae	Phataginus tetradactyla	Black-bellied Pangolin		VU (14)	Dec	
**Primates (primates)**						
Hominidae	Gorilla gorilla	Lowland Gorilla	EN	CR	Dec	112.6
Hominidae	Pan troglodytes	Chimpanzee	EN	EN	Dec	45
Hominidae	Pan paniscus	Bonobo	EN	EN	Dec	35.1
Cercopithecidae	Mandrillus sphinx	Mandrill	VU	VU	Unk	16.7
Cercopithecidae	Rhinopithecus bieti	Black Snub-nosed Monkey	EN	EN	Dec	11
Atelidae	Brachyteles arachnoides	Muriqui	EN	EN	Dec	10.5
Cercopithecidae	Pygathrix nigripes	Black-shanked Douc	EN	EN	Dec	10.3
Cercopithecidae	Macaca tonkeana	Tonkean Black Macaque	NT	VU	Dec	10
Cercopithecidae	Semnopithecus hypoleucos	Black-footed Gray Langur	NT	VU	Dec	10
Cercopithecidae	Trachypithecus auratus	Ebony Leaf Monkey	VU	VU	Dec	9.7
Cercopithecidae	Pygathrix cinerea	Gray-shanked Douc Langur	EN	CR	Dec	9.6
Cercopithecidae	Procolobus badius	Bay Colobus	EN	EN	Dec	9.5
Cercopithecidae	Procolobus gordonorum	Udzungwa Red Colobus	EN	EN	Dec	9.5
Cercopithecidae	Pygathrix nemaeus	Red-shanked Douc	EN	EN	Dec	9.4
Cercopithecidae	Macaca arctoides	Bear Macaque	VU	VU	Dec	9.4
Cercopithecidae	Rhinopithecus avunculus	Tonkin Snub-nosed Monkey	CR	CR	Dec	9.1
Atelidae	Ateles fusciceps	Black-headed Spider Monkey	CR	CR	Dec	9.1
Cercopithecidae	Colobus satanas	Black Colobus	VU	VU	Dec	9.1
Cercopithecidae	Macaca siberu	Siberut Macaque	VU	VU	Dec	9.1
Atelidae	Brachyteles hypoxanthus	Northern Muriqui	CR	CR	Dec	9
Cercopithecidae	Procolobus pennantii	Bouvier's Red Colobus	CR	CR	Dec	9
Cercopithecidae	Colobus polykomos	King Colobus	VU	VU	Unk	8.8
Atelidae	Ateles paniscus	Black Spider Monkey	VU	VU	Dec	8.7
Indriidae	Indri indri	Indri	EN	CR (14)	Dec	8.6
Atelidae	Lagothrix cana	Geoffroy'S/peruvian Woolly Monkey	EN	EN	Dec	8.6
Hylobatidae	Nomascus gabriellae	Buff-cheeked Gibbon	VU	EN	Dec	8.5
Cercopithecidae	Trachypithecus delacouri	Delacour's Langur	CR	CR	Dec	8.3
Atelidae	Oreonax flavicauda	Peruvian Yellow-tailed Woolly Monkey	CR	CR	Dec	8.2
Cercopithecidae	Trachypithecus francoisi	Francois's Langur	EN	EN	Dec	8.1
Cercopithecidae	Trachypithecus poliocephalus	Cat Ba Langur	CR	CR	Dec	8.1
Hylobatidae	Nomascus hainanus	Hainan Black Crested Gibbon	CR	CR	Stable	7.9
Cercopithecidae	Colobus vellerosus	Geoffroy's Black-and-white Colobus	VU	VU	Unk	7.7
Cercopithecidae	Trachypithecus phayrei	Phayre's Langur	EN	EN	Dec	7.7
Cercopithecidae	Simias concolor	Pig-tailed Langur	EN	CR	Dec	7.4
Cercopithecidae	Macaca nigra	Black Crested Macaque	EN	CR	Dec	7.4
Hylobatidae	Nomascus leucogenys	Northern White-cheeked Gibbon	EN	CR	Dec	7.3
Cercopithecidae	Cercocebus torquatus	Collared Mangabey	VU	VU	Dec	7.3
Atelidae	Alouatta pigra	Black Howling Monkey	EN	EN	Dec	7.2
Atelidae	Ateles chamek	Black-faced Black Spider Monkey	EN	EN	Dec	7.1

Family	Scientific Name	Common Name	Status 96	Status	Trend	Mass (kg)
Cercopithecidae	Macaca leonina	Northern Pig-tailed Macaque	VU	VU	Dec	7.1
Cercopithecidae	Cercopithecus hamlyni	Hamlyns Monkey	VU	VU	Dec	7
Cercopithecidae	Cercocebus atys	Red-capped Monkey	VU	VU	Dec	6.9
Atelidae	Ateles belzebuth	Long-haired Spider Monkey	EN	EN	Dec	6.7
Indriidae	Propithecus edwardsi	Milne-edward's Sifaka	EN	EN (14)	Dec	6.6
Cercopithecidae	Presbytis comata	Grizzled Leaf Monkey	EN	EN	Dec	6.5
Cercopithecidae	Presbytis potenziani	Long-tailed Langur	VU	EN	Dec	6.5
Hylobatidae	Hoolock hoolock	Hoolock Gibbon	EN	EN	Dec	6.5
Hylobatidae	Nomascus concolor	Black Crested Gibbon	EN	CR	Dec	6.4
Cercopithecidae	Presbytis hosei	Gray Leaf Monkey	VU	VU	Dec	6.3
Cercopithecidae	Presbytis frontata	White-faced Langur	VU	VU	Dec	6.1
Cercopithecidae	Macaca silenus	Lion-tailed Macaque	EN	EN	Dec	6
Atelidae	Lagothrix poeppigii	Poeppig's Woolly Monkey	VU	VU	Dec	5.9
Hylobatidae	Hylobates klossii	Dwarf Gibbon	VU	EN	Dec	5.8
Indriidae	Propithecus candidus	Silky Sifaka	CR	CR (14)	Dec	5.8
Hylobatidae	Nomascus siki	Southern White-cheeked Gibbon	EN	EN	Dec	5.7
Hylobatidae	Hylobates lar	Common Gibbon	VU	EN	Dec	5.6
Hylobatidae	Hylobates pileatus	Capped Gibbon	EN	EN	Dec	5.5
Cercopithecidae	Cercopithecus preussi	Preuss's Guenon	EN	EN	Dec	5.1
Cercopithecidae	Macaca pagensis	Pagai Island Macaque	EN	CR	Dec	4.5
Cercopithecidae	Cercopithecus diana	Diana Guenon	VU	VU	Dec	4.4
Indriidae	Propithecus coquereli	Coquerel's Sifaka	EN	EN (14)	Dec	4.2
Lemuridae	Varecia rubra	Red-ruffed Lemur	EN	CR (14)	Dec	3.9
Lemuridae	Varecia variegata	Black-and-white Ruffed Lemur	CR	CR (14)	Dec	3.8
Indriidae	Propithecus tattersalli	Golden-crowned Sifaka	EN	CR (14)	Unk	3.5
Pitheciidae	Cacajao calvus	Bald-headed Uacari	VU	VU	Dec	3.4
Cercopithecidae	Cercopithecus erythrotis	Red-eared Guenon	VU	VU	Dec	3.3
Pitheciidae	Cacajao hosomi	Black-headed Uacari	VU	VU	Dec	3.1
Cebidae	Cebus xanthosternos	Buff-headed Capuchin	CR	CR	Dec	3
Lemuridae	Eulemur albifrons	White-fronted Brown Lemur	VU	EN (14)	Dec	3
Lemuridae	Eulemur collaris	Collared Brown Lemur	VU	EN (14)	Dec	3
Pitheciidae	Chiropotes satanas	Bearded Saki	CR	CR	Dec	2.9
Pitheciidae	Pithecia albicans	Buffy Saki	VU	VU	Dec	2.8
Cercopithecidae	Cercopithecus dryas	Dryad Monkey	CR	CR	Unk	2.8
Pitheciidae	Chiropotes utahickae	Uta Hick's Bearded Saki	EN	EN	Dec	2.8
Daubentoniidae	Daubentonia madagascariensis	Aye-aye	NT	EN (14)	Dec	2.7
Lemuridae	Lemur catta	Ring-tailed Lemur	NT	EN (14)	Dec	2.6
Lemuridae	Eulemur macaco	Black Lemur	VU	VU (14)	Dec	2.5
Lemuridae	Eulemur rufus	Audebert's Brown Lemur	DD	VU (14)	Dec	2.4
Lemuridae	Eulemur cinereiceps	Grey-headed Lemur	EN	CR (14)	Dec	2.2
Lemuridae	Eulemur flavifrons	Blue-eyed Black Lemur		CR (14)	Dec	2.2
Lemuridae	Prolemur simus	Broad-nosed Gentle Lemur	CR	CR (14)	Dec	2
Lemuridae	Eulemur coronatus	Crowned Lemur	VU	EN (14)	Dec	1.7

Family	Scientific Name	Common Name	Status 96	Status	Trend	Mass (kg)
Lemuridae	Hapalemur alaotrensis	Alaotran Gentle Lemur	CR	CR (14)	Dec	1.6
Pitheciidae	Callicebus oenanthe	Andean Titi Monkey	EN	CR (13)	Dec	1.2
Lepilemuridae	Lepilemur microdon	Light-necked Sportive Lemur	DD	EN (14)	Dec	1
Lemuridae	Hapalemur griseus	Bamboo Lemur	VU	VU (14)	Dec	0.9
Lemuridae	Hapalemur occidentalis	Sambirano Lesser Bamboo Lemur	VU	VU (14)	Dec	0.9
Lepilemuridae	Lepilemur ruficaudatus	Lesser Weasel Lemur	DD	VU (14)	Dec	0.8
Callitrichidae	Leontopithecus caissara	Black-faced Lion Tamarin	CR	CR	Dec	0.6
Lepilemuridae	Lepilemur dorsalis	Gray's Sportive Lemur	DD	VU (14)	Dec	0.5
Lorisidae	Nycticebus pygmaeus	Lesser Slow Loris	VU	VU	Dec	0.3
Lorisidae	Loris tardigradus	Red Slender Loris	EN	EN	Dec	0.2
Cheirogaleidae	Allocebus trichotis	Hairy-eared Dwarf Lemur	DD	VU (14)	Dec	0.1
Atelidae	Ateles marginatus	White-cheeked Spider Monkey	EN	EN	Dec	
Cebidae	Cebus flavius	Blonde Capuchin	CR	CR	Dec	
Cercopithecidae	Cercocebus sanjei	Sanje Crested Mangabey	EN	EN	Dec	
Hylobatidae	Hoolock leuconedys	Eastern Hoolock	VU	VU	Dec	
Atelidae	Lagothrix lugens	Colombian Woolly Monkey	CR	CR	Dec	
Lepilemuridae	Lepilemur ankaranensis	Ankarana Sportive Lemur	EN	EN (14)	Dec	
Cercopithecidae	Macaca nigrescens	Dumoga-bone Macaque	VU	VU	Dec	
Cercopithecidae	Procolobus preussi	Preuss's Red Colobus	CR	CR	Dec	
Cercopithecidae	Rhinopithecus strykeri	Burmese Snub-nosed Monkey		CR (12)	Dec	
Tarsiidae	Tarsius tumpara	Siau Island Tarsier		CR (13)	Dec	
Cercopithecidae	Trachypithecus germaini	Germains Langur	EN	EN	Dec	
Cercopithecidae	Trachypithecus hatinhensis	Hatinh Langur	EN	EN	Dec	
Cercopithecidae	Trachypithecus laotum	Lao Langur	VU	VU	Dec	
Cercopithecidae	Trachypithecus shortridgei	Shortridges Capped Langur	EN	EN	Dec	
Lepilemuridae	Lepilemur betsileo	Betsileo Sportive Lemur	DD	EN (14)	Dec	
Lepilemuridae	Lepilemur fleuretae	Fleurete's Sportive Lemur	DD	CR (14)	Dec	
Lepilemuridae	Lepilemur grewcockorum	Grewcock's Sportive Lemur	DD	EN (14)	Dec	
Lepilemuridae	Lepilemur hubbardorum	Hubbard's Sportive Lemur	DD	EN (14)	Dec	
Lepilemuridae	Lepilemur jamesorum	James' Sportive Lemur	DD	CR (14)	Dec	
Lepilemuridae	Lepilemur milanoii	Daraina Sportive Lemur	DD	EN (14)	Dec	
Lepilemuridae	Lepilemur mittermeieri	Mittermeier's Sportive Lemur	DD	EN (14)	Dec	
Lepilemuridae	Lepilemur sahamalazensis	Sahamalaza Peninsula Sportive Lemur	DD	CR (14)	Dec	
Lepilemuridae	Lepilemur seali	Seal's Sportive Lemur	DD	VU (14)	Dec	
Lepilemuridae	Lepilemur tymerlachsoni	Hawks' Sportive Lemur		CR (14)	Dec	
Lepilemuridae	Lepilemur wrightae	Wright's Sportive Lemur	DD	EN (14)	Dec	
Cheirogaleidae	Microcebus bongolavensis	Bongolava Mouse Lemur	DD	EN (14)	Dec	
Cheirogaleidae	Microcebus danfossi	Danfoss' Mouse Lemur		EN (14)	Dec	
Cheirogaleidae	Microcebus jollyae	Jolly's Mouse Lemur	DD	EN (14)	Dec	
Indriidae	Avahi mooreorum	Masoala Woolly Lemur		EN (14)	Dec	
Lepilemuridae	Lepilemur hollandorum	Holland's Sportive Lemur		EN (14)	Dec	
Lepilemuridae	Lepilemur scottorum	Masoala Sportive Lemur		EN (14)	Dec	
Cheirogaleidae	Microcebus arnholdi	Arnhold's Mouse Lemur		EN (14)	Dec	

Family	Scientific Name	Common Name	Status 96	Status	Trend	Mass (kg)
Cheirogaleidae	Microcebus gerpi	Gerp's Mouse Lemur		CR (14)	Dec	
**Rodentia (rodents)**						
Hystricidae	Hystrix pumila	Indonesian Porcupine	VU	VU	Dec	3.5
Dasyproctidae	Dasyprocta ruatanica	Roatan Island Agouti	EN	EN	Dec	2.3
Muridae	Mallomys gunung	Alpine Woolly Rat	EN	EN	Dec	2
Muridae	Phloeomys cumingi	Southern Giant Slender-tailed Cloud Rat	VU	VU	Dec	1.8
Capromyidae	Geocapromys brownii	Brown's Hutia	VU	VU	Dec	1.5
Sciuridae	Rheithrosciurus macrotis	Tufted Ground Squirrel	VU	VU	Dec	1.4
Muridae	Crateromys schadenbergi	Giant Bushy-tailed Cloud Rat	EN	EN	Dec	1.4
Capromyidae	Plagiodontia aedium	Cuvier's Hutia	EN	EN	Dec	1.3
Muridae	Solomys ponceleti	Poncelet's Giant Rat	EN	CR	Dec	1
Sciuridae	Rubrisciurus rubriventer	Sulawesi Giant Squirrel	VU	VU	Dec	0.7
Muridae	Solomys salebrosus	Bougainville Giant Rat	VU	EN	Dec	0.4
Muridae	Paraleptomys rufilatus	Northern Hydromyine	EN	EN	Unk	0.1
Muridae	Apodemus gurkha	Himalayan Field Mouse	EN	EN	Dec	
Sciuridae	Biswamoyopterus biswasi	Namdapha Flying Squirrel	CR	CR	Dec	
Muridae	Hadromys humei	Humes Rat	EN	EN	Dec	
Diatomyidae	Laonastes aenigmamus	Laotian Rock Rat	EN	EN	Dec	
Capromyidae	Mesocapromys auritus	Eared Hutia	EN	EN	Stable	
Capromyidae	Mesocapromys sanfelipensis	Little Earth Hutia	CR(PE)	CR	Unk	
Capromyidae	Mysateles gundlachi	Chapman's Prehensile-tailed Hutia	EN	EN	Dec	
Capromyidae	Mysateles melanurus	Black-tailed Hutia		VU	Dec	
Bathyergidae	Fukomys kafuensis			VU	Dec	

References

1. Jones KE, Bielby J, Cardillo M, Fritz SA, O'Dell J, Orme CDL, et al. PanTHERIA: a species-level database of life history, ecology, and geography of extant and recently extinct mammals. Ecology. 2009 Aug 17;90(9):2648–2648.

2. Myers P, Espinosa R, Parr CS, Jones T, Hammond GS, Dewey TA. The Animal Diversity Web (online) [Internet]. 2015 [cited 2014 Feb 27]. Available from: http://animaldiversity.ummz.umich.edu

3. Arkive. ARKive - Discover the world's most endangered species [Internet]. 2015 [cited 2015 Jul 13]. Available from: http://www.arkive.org/

4. Microsoft [Internet]. Microsoft Academic Search. 2014 [cited 2014 Dec 23]. Available from: http://academic.research.microsoft.com/

5. IUCN. METADATA: Digital Distribution Maps on TheIUCN Red List of Threatened Species [Internet]. 2013. Available from: http://spatial-data.s3.amazonaws.com/groups/METADATA%20for%20Digital%20Distribution%20Maps%20of%20The%20IUCN%20Red%20List%20of%20Threatened%20Species%E2%84%A2.pdf

6. Sahr K, White D, Kimerling AJ. Geodesic discrete global grid systems. Cartogr Geogr Inf Sci. 2003;30(2):121–134.

7. Hoffmann M, Belant JL, Chanson JS, Cox NA, Lamoreux J, Rodrigues ASL, et al. The changing fates of the world's mammals. Philos Trans R Soc B Biol Sci. 2011 Aug 15;366(1578):2598–610.

8. United Nations. United Nations Statistics Division- Standard Country and Area Codes Classifications (M49) [Internet]. 2013 [cited 2013 Dec 12]. Available from: http://unstats.un.org/unsd/methods/m49/m49regin.htm

9. IUCN and UNEP-WCMC. The World Database on Protected Areas (WDPA) [On-line], July 2015 [Internet]. Cambridge, UK: UNEP-WCMC. 2015 [cited 2015 Aug 18]. Available from: http://www.protectedplanet.net/terms

10. Gardner TA, Caro TIM, Fitzherbert EB, Banda T, Lalbhai P. Conservation value of multiple-use areas in East Africa. Conserv Biol. 2007;21(6):1516–1525.
